# GWAS meta-analyses clarify the genetics of cervical phenotypes and inform risk stratification for cervical cancer

**DOI:** 10.1093/hmg/ddad043

**Published:** 2023-03-16

**Authors:** Mariann Koel, Urmo Võsa, Maarja Jõeloo, Kristi Läll, Natàlia P Gualdo, Hannele Laivuori, Susanna Lemmelä, Estonian Biobank Research Team; FinnGen, Mark Daly, Priit Palta, Reedik Mägi, Triin Laisk

**Affiliations:** Estonian Genome Centre, Institute of Genomics, University of Tartu, Tartu 51010, Estonia; Estonian Genome Centre, Institute of Genomics, University of Tartu, Tartu 51010, Estonia; Estonian Genome Centre, Institute of Genomics, University of Tartu, Tartu 51010, Estonia; Estonian Genome Centre, Institute of Genomics, University of Tartu, Tartu 51010, Estonia; Estonian Genome Centre, Institute of Genomics, University of Tartu, Tartu 51010, Estonia; Institute for Molecular Medicine Finland, FIMM, HiLIFE, University of Helsinki, Helsinki 00014, Finland; Faculty of Medicine and Health Technology, Department of Obstetrics and Gynecology, Tampere University Hospital and Tampere University, Tampere 33520, Finland; Medical and Clinical Genetics, University of Helsinki and Helsinki University Hospital, Helsinki 00014, Finland; Institute for Molecular Medicine Finland, FIMM, HiLIFE, University of Helsinki, Helsinki 00014, Finland; Institute for Molecular Medicine Finland, FIMM, HiLIFE, University of Helsinki, Helsinki 00014, Finland; Estonian Genome Centre, Institute of Genomics, University of Tartu, Tartu 51010, Estonia; Institute for Molecular Medicine Finland, FIMM, HiLIFE, University of Helsinki, Helsinki 00014, Finland; Estonian Genome Centre, Institute of Genomics, University of Tartu, Tartu 51010, Estonia; Estonian Genome Centre, Institute of Genomics, University of Tartu, Tartu 51010, Estonia

## Abstract

Genome-wide association studies (GWAS) have successfully identified associations for cervical cancer, but the underlying mechanisms of cervical biology and pathology remain uncharacterised. Our GWAS meta-analyses fill this gap, as we characterise the genetic architecture of cervical phenotypes, including cervical ectropion, cervicitis, cervical dysplasia, as well as up to 9229 cases and 490 304 controls for cervical cancer from diverse ancestries. Leveraging the latest computational methods and gene expression data, we refine the association signals for cervical cancer and propose potential causal variants and genes at each locus. We prioritise *PAX8/PAX8-AS1*, *LINC00339*, *CDC42*, *CLPTM1L*, *HLA-DRB1* and *GSDMB* as the most likely candidate genes for cervical cancer signals, providing insights into cervical cancer pathogenesis and supporting the involvement of reproductive tract development, immune response and cellular proliferation/apoptosis. We construct a genetic risk score (GRS) that is associated with cervical cancer [hazard ratios (HR) = 3.1 (1.7–5.6) for the top 15% vs lowest 15% of individuals], and with other HPV- and immune-system-related diagnoses in a phenome-wide association study analysis. Our results propose valuable leads for further functional studies and present a GRS for cervical cancer that allows additional risk stratification and could potentially be used to personalise the conventional screening strategies for groups more susceptible to cervical cancer.

## Introduction

Cervical cancer (CC) is one of the most common cancer types in women, with more than 28 000 and 311 000 women dying from the disease in Europe and worldwide every year, respectively (1). Although the development of CC is initiated by human papillomavirus (HPV) high-risk subtype infection, host genetics also influences its development and prognosis. Previous family-based studies have estimated the heritability of CC to be 13–29% (2,3) whereas the array-based heritability estimate is 7% (2–12%) (4), and recent large genome-wide association studies (GWAS) have also increased the number of loci reported for CC (4,5). However, these genetic associations are merely the first step in mapping genetic susceptibility and biology and are, therefore, on their own often insufficient to connect the variant to function and causal mechanisms. Thus, the underlying carcinogenic mechanisms and molecular changes in CC are still not entirely understood (6), nor has the applicability of genetic risk scores (GRS) in the context of CC been fully explored.

Similarly, not much is known about the genetic factors modifying other cervical phenotypes, such as cervical ectropion, a benign condition where the columnar epithelium of the cervical canal is turned outwards and exposed to the vaginal environment (7); cervicitis, inflammation of the uterine cervical epithelium, most commonly caused by sexually transmitted pathogens, such as *Chlamydia trachomatis*, *Neisseria gonorrhoea* and *Mycoplasma genitalium* (8); and cervical dysplasia, a precancerous condition with varying severity, characterised by abnormal growth of the cervical epithelium. These phenotypes all represent partially overlapping conditions of the uterine cervix with similar symptoms. Without knowing the full spectrum of genetic determinants for cervical biology and its disorders, it is difficult to evaluate if the findings of CC GWAS are specific to (cervical) cancer, part of cervical biology or also relevant to other conditions, such as ectropion or cervicitis. So far, GWAS have been conducted only for severe cervical dysplasia or cervical cancer, but the field lacks studies for benign phenotypes. Therefore, the more we understand about the genetic regulation of cervical development and function, the better equipped we are to investigate the molecular basis of CC formation and offer sufficient risk predictions.

Here we use data from Estonian Biobank (EstBB) and the FinnGen study to dissect the genetic architecture of cervical phenotypes in a sample set including cases of cervical ectropion (*n* = 10 162), cervicitis (*n* = 19 285) and cervical dysplasia (*n* = 14 694). We then explore their genetic overlap with CC by combining all publicly available datasets in the largest multi-ancestry GWAS meta-analysis of CC to date, with 9229 CC cases and 490 304 controls. Leveraging the latest computational methods and gene expression data, we refine the association signals for CC and propose potential causal variants and genes at each locus for functional follow-up. Finally, we construct a GRS for cervical cancer, assess its risk stratification ability and present the pleiotropic phenomic network associated with genetic risk for CC.

## Results

First, to determine the genetic factors associated with cervical phenotypes, we conducted GWAS for cervical ectropion, cervicitis and dysplasia using data for 92 042 female individuals from the EstBB. Next, the results of these analyses were meta-analysed together with the corresponding summary statistics from the FinnGen study, using FinnGen R5 release data with up to 112 951 Finnish female individuals. The resulting meta-analysis included 10 162 women with cervical ectropion, 19 285 with cervicitis, 14 694 with cervical dysplasia and up to 193 452 female controls of European ancestry (EUR).

### GWAS meta-analyses for cervical ectropion, cervicitis and dysplasia

We identified one genome-wide significant (*P* < 5 $\times$ 10^−8^) locus for both cervical ectropion and cervicitis (Supplementary Material, Fig. S1), and five signals for cervical dysplasia (Table 1; Supplementary Material, Fig. S2). Altogether, the number of analysed markers in the meta-analysis was up to 11 043 697. All the reported genetic variants show at least nominal significance in both analysed cohorts (Table 1).

**Table 1:** GWAS meta-analyses result (EstBB and FinnGen). Genetic variants associated with cervical ectropion, cervicitis and dysplasia

Phenotype	chr:pos (b37)	Variant (EA)		Meta-analysis	EstBB	FinnGen	Nearest gene^a^
Ectropion	2:113984033	rs3748916 (A)	*P*-value OR (95% CI) EAF	5.1 $\times$ 10^−16^ 1.14 (1.10–1.17) 0.41	8.3 $\times$ 10 ^−13^ 1.14 (1.10–1.17) 0.42	0.208 1.08 (0.94–1.24) 0.40	*PAX8*
Cervicitis	2:113975110	rs1049137 (G)	*P*-value OR (95% CI) EAF	3.9 $\times$ 10^−10^ 0.92 (0.91–0.94) 0.25	6.3 $\times$ 10^−10^ 0.92 (0.91–0.94) 0.26	0.26 0.94 (0.85–1.04) 0.25	*PSD4*
Dysplasia	2:113975110	rs1049137 (G)	*P*-value OR (95% CI) EAF	6.4 $\times$ 10^−9^ 0.92 (0.91–0.94) 0.26	0.001 0.94 (0.91–0.98) 0.26	1.6 $\times$ 10^−8^ 0.86 (0.81–0.91) 0.25	*PSD4*
	2:159629994	rs12611652 (A)	*P*-value OR (95% CI) EAF	3.2 $\times$ 10^−9^ 0.92 (0.91–0.94) 0.54	2.1 $\times$ 10^−5^ 0.93 (0.90–0.97) 0.52	1.9 $\times$ 10^−5^ 0.90 (0.87–0.94) 0.56	*DAPL1*
	5:1311693	rs6866294 (C)	*P*-value OR (95% CI) EAF	2.1 $\times$ 10^−9^ 1.08 (1.06–1.10) 0.57	1.0 $\times$10^−5^ 1.07 (1.03–1.12) 0.60	3.3 $\times$ 10^−5^ 1.11 (1.06–1.15) 0.54	*TERT*
	6:31322047	rs1053726 (G)	*P*-value OR (95% CI) EAF	9.1 $\times$ 10^−9^ 0.91 (0.88–0.95) 0.19	5.5 $\times$ 10^−4^ 0.93 (0.90–0.97) 0.18	3.9 $\times$ 10^−7^ 0.87 (0.82–0.92) 0.21	*HLA-B*
	6:32611759	rs36214159 (G)	*P*-value OR (95% CI) EAF	1.6 $\times$ 10^−22^ 0.79 (0.76–0.83) 0.09	3.6 $\times$ 10^−9^ 0.85 (0.80–0.90) 0.09	1.3 $\times$ 10^−18^ 0.69 (0.64–0.75) 0.09	*HLA-DQA1*

^a^Nearest gene based on Open Targets Genetics portal.

Notably, all three analysed phenotypes showed significant association with a locus on chromosome 2 near the *PAX8* gene and its antisense RNA *PAX8-AS1*. PAX8 is a transcription factor known to be relevant for genital tract development.

Furthermore, we observed additional four genome-wide significant signals for cervical dysplasia—two in the human leukocyte antigen (HLA) region on chromosome 6 (rs1053726, *P* = 9.1 $\times$ 10^−9^, rs36214159, *P* = 1.6 $\times$ 10^−22^), one on chromosome 2 (rs112611652, *P* = 3.2 $\times$ 10^−9^) near *DAPL1* and one on chromosome 5 (rs6866294, *P* = 2.1 $\times$ 10^−9^), downstream *CLPTM1L.*

### GWAS meta-analysis for cervical cancer

To determine the locus-level genetic overlap between cervical phenotypes and CC, we conducted another GWAS analysis with the EstBB data for CC (n_cases_ = 748) and combined the results with publicly available GWAS summary statistics resulting in the largest GWAS meta-analysis for CC to date. We used data from FinnGen release R4 (n_cases_ = 1313; https://r4.finngen.fi/pheno/C3_CERVIX_UTERI), Rashkin *et al.* 2020 (4) (n_cases_ = 6563) and Biobank Japan (n_cases_ = 605; http://jenger.riken.jp:8080/pheno/Cervical_cancer), resulting in a total of 8624 cases and 400 573 controls for the EUR meta-analysis, and 9229 CC cases and 490 304 controls in the multi-ancestry meta-analysis.

**Figure 1:**
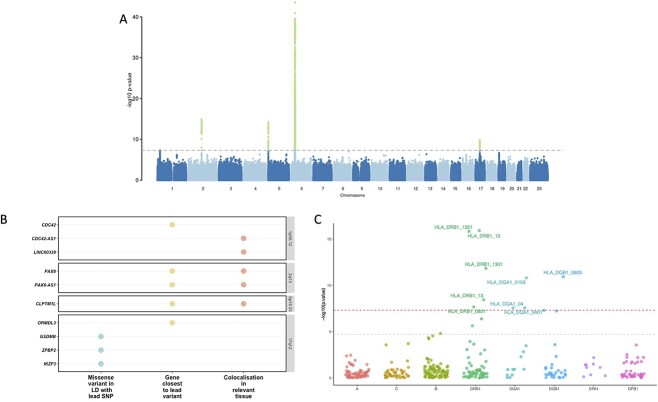
Genome wide association study for cervical cancer, gene prioritisation and HLA fine-mapping. (**A**) Manhattan plot of cervical cancer GWAS meta-analysis. (**B**) Visualised evidence for cervical cancer GWAS meta-analysis candidate gene mapping (showing only genes with at least one level of evidence). We considered the following criteria when selecting the most likely candidate genes—(a) whether the lead signal is in LD (*r*^2^ > 0.6) with a coding variant in any of the nearby genes, (b) which is the closest gene to GWAS lead variant in each locus and (c) is there significant (PP > 0.8) colocalisation in relevant tissues (tissues similar to female reproductive tract tissues based on cellular composition and gene expression (vagina, uterus, oesophagus mucosa and gastro-oesophageal junction, sigmoid colon, skin, salivary gland and tibial nerve). (**C**) HLA alleles associated with cervical dysplasia. The *y*-axis shows the -log10 *P*-values from analysis of 10 446 cases and 81 586 controls in the EstBB using SAIGE. The red dashed line represents the genome-wide significance threshold (*P* < 5 $\times$10^−8^), whereas the grey line represents the *P*-value threshold adjusted for the number of tested alleles (*P* < 2.0$\times$10^−5^).

As a result, we identified five loci associated with CC (Table 2; Supplementary Material, Fig. S3): 1p36.12 (rs2268177, *P* = 3.08 $\times$ 10^−8^), 2q13 (rs4849177, *P* = 9.36 $\times$ 10^−15^), 5p15.33 (rs27069, *P* = 1.31 $\times$ 10^−14^), 17q12 (rs12603332, *P* = 1.18 $\times$ 10^−9^) and in the HLA region on 6p21.32 (multi-ancestry meta-analysis: rs35508382, *P* = 1.04 $\times$10^−39^; EUR analysis: rs28718232, *P* = 2.55 $\times$ 10^−44^), with similar effect estimates in EUR and Biobank Japan datasets (Table 2). We then proceeded to define the most likely causal single nucleotide polymorphisms (SNPs) and the most likely causal gene at each associated locus using the EUR meta-analysis results. For this analysis, we excluded the HLA region, for which we conducted separate signal fine mapping (see below). We considered the following criteria when selecting the most likely candidate genes (Fig. 1)—(a) whether the lead signal is in linkage disequilibrium (LD) with a coding variant in any of the nearby genes, (b) which is the closest gene to the GWAS lead variant in each locus and (c) is there significant (posterior probability (PP) > 0.8) colocalisation in relevant tissues (tissues similar to female reproductive tract tissues based on cellular composition and gene expression). For credible set variants, we highlighted those that have a larger regulatory potential based on the HeLa cell line data (Supplementary Material, Fig. S4 and Supplementary Material, Table S1).

**Table 2:** Genetic variants associated with cervical cancer in a total of 8624 cases and 400 573 controls of European ancestry (EstBB, FinnGen, UKBB+Kaiser Permanente) and 9229 cervical cancer cases and 490 304 controls in the multi-ancestry meta-analysis (EstBB, FinnGen, UKBB+Kaiser Permanente and Biobank Japan)

Variant (EA)	chr:pos (b37)	*P*-value European ancestry meta-analysis	*P*-value multi-ancestry meta-analysis	OR (95% CI) European ancestry meta-analysis	OR (95% CI) Biobank Japan dataset	EAF Eur	EAF Biobank Japan	Nearest gene^a^
rs2268177 (T)	1:22415410	3.8 $\times$ 10^−8^	3.1 $\times$ 10^−8^	1.12 (1.07–1.16)	1.13 (1.01–1.27)	0.18	0.54	*CDC42*
rs4849177 (C)	2:113982584	1.3 $\times$ 10^−15^	9.4 $\times$ 10^−15^	0.87 (0.85–0.91)	0.95 (0.84–1.07)	0.39	0.35	*PAX8*
rs27069 (T)	5:1347128	6.1$\times$ 10^−15^	1.3 $\times$ 10^−14^	0.88 (0.85–0.91)	0.83 (0.71–0.97)	0.43	0.14	*CLPTM1L*
rs35508382 (G)^b^	6:32593144	8.4 $\times$ 10^−40^	1.0 $\times$ 10^−39^	0.67 (0.63–0.71)	0.82 (0.70–0.97)	0.10	0.14	*HLA-DQA1*
rs12603332 (C)	17:38082807	1.6 $\times$ 10^−10^	1.2 $\times$ 10^−9^	0.90 (0.88–0.93)	0.96 (0.85–1.09)	0.49	0.72	*ORMDL3*

^a^Nearest gene based on Open Targets Genetics portal.

^b^For European ancestry analysis, we detected on chr6 HLA-region lead signal as rs28718232, *P* = 2.55 $\times$ 10^−44^, but it was not a lead signal in the Biobank Japan dataset and therefore we present the common variant rs35508382 for both meta-analyses.

The lead variant on 1p36.12 (rs2268177) is in the intron of *CDC42*, downstream *WNT4*. Colocalisation analysis showed that CC GWAS association signal colocalises with *CDC42*, *CDC42-AS1* and *LINC00339* expression/transcription events in several tissues and cell types (Supplementary Material, Table S2) and corresponding credible sets included 40 variants (Supplementary Material, Table S3). *CDC42-AS1* and *LINC00339* were also prioritised based on the colocalisation signal in trait-relevant tissue (Fig. 1), with high colocalisation probability (PP4 = 0.94) between the GWAS signal and *CDC42-AS1* gene expression in oesophagus mucosa, and between the GWAS signal and *LINC00339* transcripts ENST00000635675 and ENST00000434233 in GTEx skin dataset. In both colocalisations, rs2473290 (in the intron of *CDC42-AS1*) explains most of the shared association (posterior inclusion probability 0.95–0.99). Of the other credible set variants, rs3768579 and rs3754496 are located in transcription start site (TSS) flanking regions of *LINC00339* and *CDC42* in HeLa cells, whereas rs72665317 and rs10917128 overlap with enhancer marks (Supplementary Material, Table S1 and Supplementary Material, Fig. S4). *LINC00339* has a known role in promoting the proliferation of several cancers (9–11), while there is also evidence to link *CDC42* expression with CC invasion and migration (12). The region has been previously associated with uterine fibroids, endometriosis, endometrial cancer (13), epithelial ovarian cancer, gestational age and bone mineral density (Supplementary Material, Table S4, Supplementary Material, Fig. S5).

As with other cervical phenotypes, we observed a significant association on chromosome 2, where the lead variant (rs4849177) is in an intronic region of *PAX8*. The GWAS signal colocalises with the expression of *PAX8* and its potential regulator, *PAX8-AS1*, in several tissues and cell types, and the credible set included 29 variants. Of the credible set variants, rs1015753 overlaps with a TSS flanking region in HeLa cells, whereas another six variants overlap with regulatory enhancer elements (Supplementary Material, Table S1). Colocalisation signals for *PAX8* and *PAX8-AS1* were also observed in several relevant tissues, including the vagina (Supplementary Material, Table S2), where the credible set included 13 variants (Supplementary Material, Table S3), two of them overlapping with enhancer elements.

We compared the signal in the 2q13 locus across the analysed cervical phenotypes (Supplementary Material, Figs 6 and 7; Supplementary Material, Table S5) and found that the lead signals for ectropion (rs3748916) and cervicitis/dysplasia (rs1049137) are not in high linkage disequilibrium (*r*^2^ = 0.27, 1000G p3v5 EUR), indicating independent or partly independent signals in the same region. The CC lead signal was moderately correlated (*r*^2^ = 0.45–0.53, EUR) with cervicitis/dysplasia and ectropion signals, respectively. This is supported by the fact that although the sets of most likely causal variants mostly overlapped for cervicitis, dysplasia and cancer, the credible set variants seem to be different for ectropion.

The signal on chromosome 5 (lead variant rs27069) locates upstream of *CLPTM1L* and overlaps with the TSS in HeLa cells (Supplementary Material, Fig. S4). Numerous colocalisations with different *CLPTM1L* quantitative trait locus (QTL) events were observed, including in skin and gastroesophageal junction datasets (Supplementary Material, Table S2). *CLPTM1L* is a membrane protein and its overexpression in cisplatin-sensitive cells causes apoptosis. Polymorphisms in this region have been reported to increase susceptibility to cancer, including lung, pancreatic and breast cancers (Supplementary Material, Table S4). Variants in the credible set overlap with active TSS, as well as with several enhancer and zinc finger (ZNF) repeat marks in the *CLPTM1L* gene (Supplementary Material, Fig. S4).

On chromosome 17, the lead signal (rs12603332) is in high LD (*r*^2^ > 0.8) with a splice acceptor variant (rs11078928) in *GSDMB*. GSDMB belongs to the family of gasdermin-domain-containing proteins. Members of this family regulate apoptosis in epithelial cells and are linked to cancer (14). GSDMB has also been linked with invasion and metastasis in breast cancer cells (15) and in CC (16). Specifically, the splice variant rs11078928 deletes exon 6, which encodes 13 amino acids in the critical N-terminus, and therefore, abolishes the pyroptotic activity (pyroptosis is a type of cell death) of the GSDMB protein (17). This region has been previously associated with asthma, inflammatory bowel disease, ulcerative colitis, Crohn’s disease, multiple sclerosis, primary biliary cholangitis, rheumatoid arthritis and other disorders with an immune aetiology, but also with CC (18).

Given the similarity in signals identified for cervical dysplasia and CC (Tables 1 and 2), we jointly analysed the GWAS results for dysplasia and cancer and identified an additional signal on chromosome 19 (rs425787, *P* = 3.5 $\times$10^−8^, Supplementary Material, Fig. S8) that remained below the significance threshold in the CC analysis alone (*P* = 2.1$\times$10^−7^). Since this locus was not significant in the CC meta-analysis, it was not included in the colocalisation and fine-mapping analyses. This association signal overlaps with enhancer histone marks in HeLa cervical carcinoma cell line (Supplementary Material, Fig. S8) and is in the 3′ regions of *CD70*. CD70 is a cytokine with an important role in T-cell immunity during the antiviral response, and its high expression has been associated with a favourable outcome in CC patients (19).

### Dysplasia signals stratified by dysplasia severity and in cervical cancer

**Figure 2:**
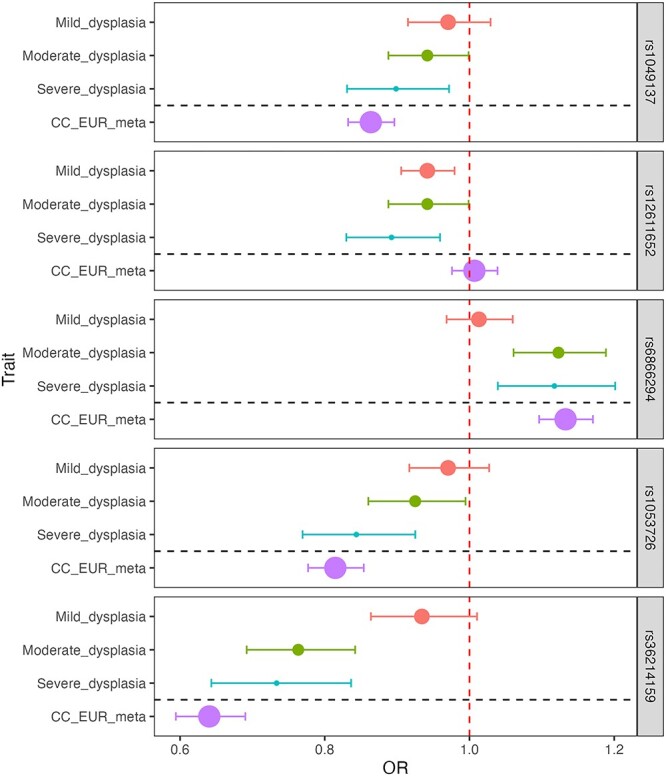
Dysplasia lead signals in different dysplasia stages and cervical cancer in European ancestry analyses. Data are presented as odds ratios (dot) and 95% confidence intervals (error bars) originating from GWAS analysis. The size of the dot is proportional to the effective sample size (calculated as 4/((1/N_cases) + (1/N_controls)). The red dashed line represents the line of no effect.

We stratified the dysplasia phenotype to evaluate the meta-analysis effect sizes (odds ratios) about pathology severity. Figure 2 shows the effect estimates in dysplasia subphenotypes and CC meta-analysis from EUR. In general, odds ratios correlated with the degree of pathology, although there was an overlap in confidence intervals (Fig. 2). An interesting exception seems to be rs12611652 near *DAPL1*, which is associated with different cervical dysplasia subphenotypes, but not with CC. *DAPL1* is expressed in the epithelium and may play a role in the early stages of epithelial differentiation or apoptosis and is a suppressor of cell proliferation in retinal pigment epithelium (20). The GWAS signal colocalises with *PKP4* expression in gastro-oesophageal junction tissue, with three SNPs in the credible set (Supplementary Material, Table S6). PKP4 regulates junctional plaque organisation, cadherin function and cell adhesion.

All CC lead signals were at least nominally significant (*P* < 0.05) in cervical dysplasia analysis, rs4849177 and rs35508382 were also genome-wide significant (Supplementary Material, Table S7), confirming the overlap of genetic risk factors for cervical dysplasia and cancer.

### Gene-based testing in cervical cancer

The results from gene-based testing largely mirror the results of the single variant analysis. Apart from numerous genes on chromosome 6, *CLPTM1L*, *PAX8*, *PSD4*, *GSDMB*, *ORMDL3*, *ZPBP2*, *CD70* and *SKAP1* passed the significance level threshold (*P* < 2.5 $\times$10^−6^, Supplementary Material, Table S8), with the association with *SKAP1* being a novel finding compared to single variant analysis. SKAP1 is a T-cell adaptor protein with a critical role in coupling T-cell antigen receptor stimulation to the activation of integrins. The *SKAP1* locus has been previously associated with ovarian cancer (21).

### Look-up of variants previously associated with cervical cancer

Of the 170 variants with an rs-number extracted from the GWAS catalogue as (potentially) associated (*P*-value < 9 $\times$10^−6^) with CC, 55 were present in all four of the cohorts included in the multi-ancestry meta-analysis. Of these, 34 had a *P*-value < 9.1 $\times$10^−4^ (Supplementary Material, Table S9), which is the Bonferroni corrected threshold of significance (0.05/55). In the EUR analysis, 64/170 variants were present and 19 passed the Bonferroni corrected threshold of significance (0.05/64 = 7.8 $\times$ 10^−4^), including variants in/near *PAX8*, *MUC21/MUC22*, the *HLA* gene cluster and *GSDMB*.

**Figure 3:**
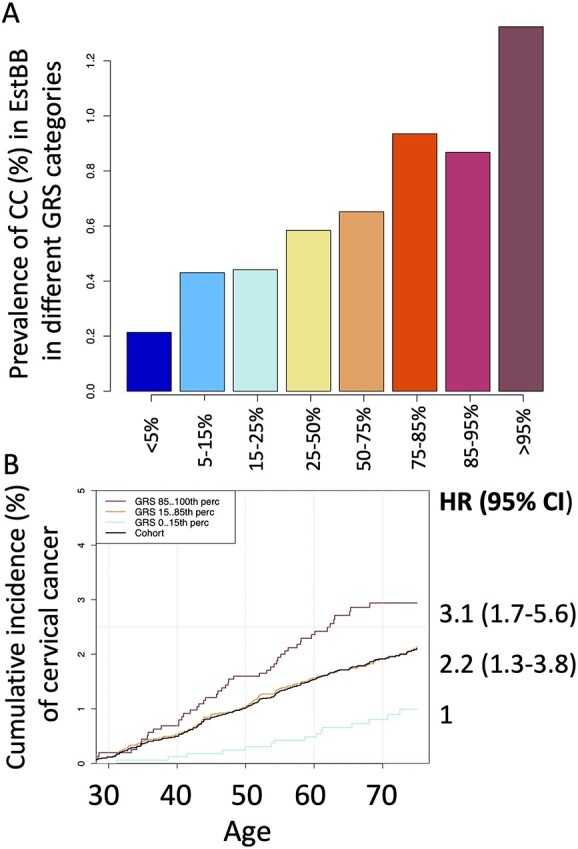
Prevalence of cervical cancer in different genetic risk categories (top) and cumulative incidence in % according to genetic risk (bottom) in EstBB. Cumulative incidence accounting for competing risk in three GRS categories, in women up to 70 years. The black line represents the cohort average.

### HLA fine-mapping

Since both cervical dysplasia and CC show an association signal in the HLA region, we used the larger cervical dysplasia dataset in EstBB to further map the cervical dysplasia association signal in the HLA region (Fig. 1). HLA-DRB1*1201 [*P* = 1.2 $\times$ 10^−16^, odds ratio (OR) = 0.74 (0.68–0.79)], HLA-DRB1*1301 [*P* = 1.5 $\times$ 10^−11^, OR = 0.82 (0.78–0.87)], HLA-DQB1*0603 [*P* = 1.2 $\times$ 10^−11^, OR = 0.83 (0.79–0.88)], HLA-DQA1*0103 [*P* = 1.6 $\times$10^−11^, OR = 0.83 (0.79–0.88)], HLA-DRB1*0801 [*P* = 2.2 $\times$10^−8^, OR = 1.20 (1.12–1.27)] and HLA-DQA1*0401 [*P* = 2.8 $\times$10^−8^, OR = 1.19 (1.12–1.27)] alleles passed the genome-wide significance threshold. These results are in line with previous studies in CC—*HLA*-DRB1*1301 and DQB1*0603 alleles are associated with decreased risk (22–25), and more broadly, the *HLA*-DRB1*1301–*HLA*-DQA1*0103–*HLA*-DQB1*0603 haplotype has been shown to protect against CC (26). *HLA*-DRB1*0801 and *HLA*-DQA1*0401 are in strong LD with *HLA*-DQB1*0402 (*P* = 5.2 × 10^−8^) and have been associated with autoimmune disease, including type 1 diabetes and systemic lupus erythematosus (27).

### Genetic correlations

We evaluated pairwise genetic correlations (r_g_) between CC and 33 selected traits from LD Hub (28). We found two significant [False discovery rate (FDR) < 0.05] genetic correlations—the age at first birth (r_g_ = −0.37, se = 0.08) and former versus current smoking status (r_g_ = −0.45, se = 0.14). Several other traits reflective of smoking behaviour (incl. lung cancer) were also nominally significant (Supplementary Material, Table S10).

### Genetic risk score for cervical cancer

Evaluating a total of ten risk score profiles, we found the best performing score had an OR = 1.45 (95% confidence interval (CI) 1.32–1.59, *P* = 1.68 $\times$ 10^−14^) for discriminating between CC case/control status in the discovery stage (Supplementary Material, Fig. S9). CC prevalence in EstBB according to genetic risk categories can be seen in Figure 3.

The HLA fraction of the score had an OR = 1.35 (1.23–1.48) and the non-HLA fraction OR = 1.25 (1.14–1.37), indicating that the majority of the predictive power comes from the *HLA* region, with marginal contribution from the rest of the genome.

We then evaluated the performance of the best-performing GRS in the validation set, consisting of incident CC cases (*n* = 235) and controls (*n* = 127 878). In the validation set, the risk increased 1.33-fold per 1 standard deviation (SD) increase of risk score and the continuous distribution of GRS showed a c-statistic of 0.61 (SD = 0.02). The C-statistic gives the probability that a randomly selected individual who experienced cervical cancer had a higher risk score than an individual who did not have cervical cancer. The C-statistic is equal to the area under curve (AUC), and an AUC or C-statistic 0.60–0.75 means possibly helpful discrimination (29). Then, we divided the GRS into following categories: < 5%, 5–15%, 15–25%, 25–50%, 50–75%, 75–85%, 85–95%, > 95%, and < 15%, 15–85%, > 85%. Cumulative incidence of CC according to genetic risk category while accounting for competing events (death) can be seen in Figure 3. For women in the top 15% risk group, the CC rate was 3.1 times (95% CI 1.7–5.6) as great as that for the individuals in the lowest 15%.

**Figure 4:**
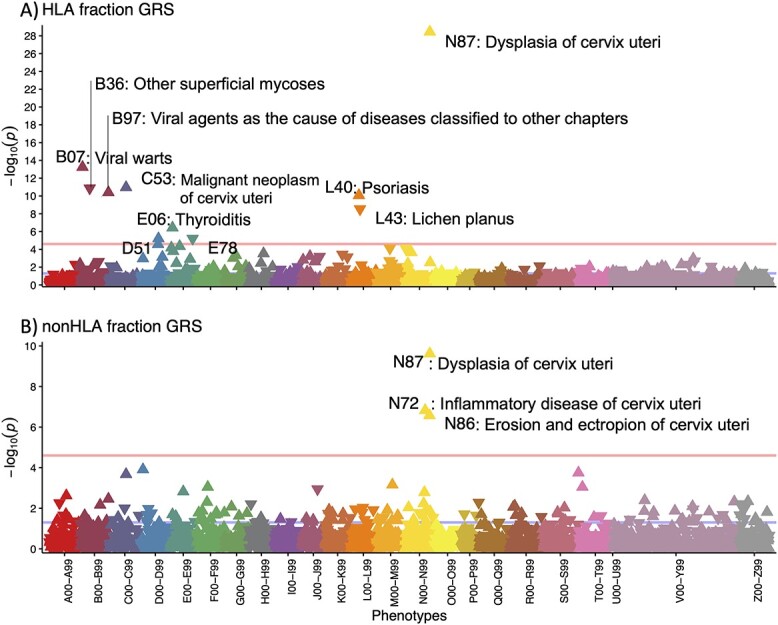
pheWAS results for association with cervical cancer risk score HLA (top) and non-HLA (bottom) fractions. Each triangle in the plot represents one ICD10 main code and the direction of the triangle represents the direction of effect—upward-pointing triangles show an increased probability of a diagnosis code in individuals with higher GRS. Pink line—Bonferroni-corrected significance level (*P* = 2.5 $\times$ 10^−5^).

To interpret our findings and assess the overlap with other phenotypes, we used GRSs in phenome-wide association study (pheWAS) analyses (Fig. 4). Beyond associating with CC, the higher genetic risk was also associated with an increased risk of dysplasia, viral warts and diseases with a suspected autoimmune aetiology: thyroiditis and psoriasis (Table 3). At the same time, a higher CC GRS was associated with a lower risk of *lichen planus*, a chronic inflammatory skin condition affecting the skin and mucosal surfaces, and other superficial mycoses. In sex-stratified analyses, the female-only results mirrored those of the overall analysis, whereas in the male-only analysis, the GRS was associated with viral warts.

**Table 3:** Results of the phenome-wide association study for cervical cancer genetic risk score. Bonferroni correction was applied to select statistically significant associations (number of tested ICD main codes—2001, corrected *P*-value threshold 0.05/2001 = 2.5 $\times$ 10^-5^

Diagnosis	*P*-value				
	Overall	Female-only	Male-only	HLA fraction	non-HLA-fraction
C53 Malignant neoplasm of cervix uteri	2.1 $\times$10^−14^	2.1 $\times$10^−14^	NA	1.1 $\times$10^−11^	2.1 $\times$10^−4^
D06 Carcinoma in situ of cervix uteri	8.9 $\times$10^−7^	3.2 $\times$10^−7^	NA	1.1 $\times$10^−3^	1.2 $\times$10^−4^
N87 Dysplasia of cervix uteri	2.6 $\times$10^−37^	2.6 $\times$10^−37^	NA	3.6 $\times$10^−29^	2.4 $\times$10^−10^
B07 Viral Warts	1.9 $\times$10^−7^	5.9 $\times$10^−3^	1.5 $\times$10^−7^	5.7 $\times$10^−14^	0.17
B36 Other superficial mycoses	1.7 $\times$10^−7^	2.5 $\times$10^−7^	0.09	1.3 $\times$10^−11^	0.76
B97 Viral agents as the cause of diseases classified elsewhere	2.0 $\times$ 10^−12^	1.4 $\times$ 10^−13^	0.42	4.0 $\times$10^−11^	3.5 $\times$10^−3^
E06 Thyroiditis	4.8 $\times$10^−8^	5.5 $\times$10^−7^	0.02	3.7 $\times$10^−7^	0.02
L40 Psoriasis	1.1 $\times$10^−5^	1.6 $\times$10^−4^	0.02	8.5 $\times$10^−11^	0.18
L43 Lichen planus	6.4$\times$10^−8^	4.9$\times$10^−5^	2.5$\times$10^−4^	2.9 $\times$10^−9^	0.27

When we explored the phenotypic associations with the HLA-partitioned GRS, we expectedly found that the HLA GRS was associated with diagnoses where HLA plays a role in the etiopathogenesis, whereas non-HLA GRS was associated only with different cervical phenotypes, such as cervical dysplasia. In the non-HLA GRS pheWAS, CC codes (C53 and D06) were nominally significant but did not pass the multiple testing threshold.

## Discussion

Here, we present the results from the largest multi-ancestry GWAS meta-analysis of CC and other cervical phenotypes, encompassing up to 9229 CC cases and 490 304 controls. Our analysis resulted in five significant loci and estimated non-HLA heritability of 4.75% for cervical cancer. This is in line with previously reported array-based heritability [7% (95%CI 2–12%)] (4), but due to the methodology used, these estimates do not include the contribution from the HLA region. Previous family/registry studies have reported slightly higher heritabilities (13–29%) (2,3), suggesting that a considerable portion of genetic variation may remain unaccounted for in the current heritability estimates and potentially also in GWAS studies. Several aspects of our study once again highlight the central role of HPV infection and the host immune response to the infection in cervical cancer development.

The GWAS sample size and precisely defined cases are relevant for more accurate genetic predictors. We observed that identified genetic associations were very similar in both dysplasia and cancer, and mirrored closely the results from a recent joint analysis of severe dysplasia and CC (5). This indicates that further GWAS studies could include both phenotypes to increase the study power. By analysing the genetics of cervical ectropion and cervicitis in addition to dysplasia and cancer, we conclude that *PAX8/PAX8-AS1* appears to have a dual role in cervical biology: PAX8 signalling is not only important for female genital system development but could enhance the proliferation of tumour cells (30).

Although previous studies have reported relevant association signals (5), we also evaluated the colocalisation of GWAS signals from different traits with expression quantitative trait locus (eQTL) signals, which gave valuable information on potential shared causal variants and trait-relevant genes, providing the link between genetics, gene expression and disease risk. Overall, our results replicate the CC associations near *PAX8* (4,5), *CLPTM1L* (5), *HLA-DRB1* (22), *HLA-B* (5) and *GSDMB* (18). The association with chromosome 1 is novel, although the region is a known risk locus for other gynaecological problems. Our results support *LINC00339* and *CDC42/CDC42-AS1* as the most likely candidate genes in this locus, which is in line with evidence from other cancers (9–12). Previous studies have shown that knocking down *LINC00339* expression leads to increased *CDC42* expression (31), which is supported by data from eQTLs—variants associated with increased expression of *LINC00339* have an opposite effect on *CDC42* expression (32).

The GRS constructed based on our analyses shows a strong association with CC in the EstBB. Additional analyses show that a large part of the predictive power comes from associations in the HLA region, which is not surprising given the major role of HPV infection and HLA-mediated immune response in the pathogenesis of cervical malignancy. Although further analyses are needed, this might indicate that in the context of CC, testing of HLA alleles might be largely sufficient for risk profiling. On the other hand, this also underlines the importance of considering disease biology when constructing GRS and using appropriate LD reference panels, since many commonly used LD references offer different coverage for the HLA region (33) and may not capture the correct population-specific LD structure, therefore, leading to underperformance of tested GRS. A previous study exploring the performance of GRS in CC found that women in the highest 5% have approximately 22% risk of developing cervical neoplasia (34); however, in this study the cases and controls originated from different populations, which can lead to unwanted stratification and differences in allele frequencies, making it difficult to compare with our results. More recent studies (4,35) constructed a GRS for CC using ten variants (all on chr6) with corresponding ORs from previous literature and validated its association with CC with an OR = 1.22 per SD increase in the GRS. These results indicate that a GRS for CC captures the genetic risk well and might be useful for research and screening purposes, either for enhancing the target population or timing of screening programs (36).

A pheWAS with the GRS demonstrated a positive association with other diagnoses associated with HPV infection (cervical dysplasia and viral warts) and HLA involvement (psoriasis and thyroiditis), and a negative association with *lichen planus* and superficial mycoses. The aetiology of *lichen planus* is somewhat poorly studied and potentially involves autoimmune aetiology, but a decreased incidence of CC has been demonstrated in *lichen sclerosus* (37)*,* another skin disease with suspected autoimmune aetiology and preference for the genitalia (38). Although the genetics of *lichen planus* has not been studied thoroughly, our results suggest that in terms of HLA associations, cervical malignancy and *lichen planus* are mirror phenotypes.

It has been suggested that persistent HPV infection and CC are more common in women with autoimmune disease (39,40), partly because of systemic immunosuppressive drugs prescribed to these women (39). However, our results suggest that shared genetic predisposition may also play a role, as the combination of HLA alleles associated with the risk of cervical dysplasia has also been associated with autoimmune diseases. This is supported by the association we see between the CC GRS and autoimmune conditions (psoriasis and thyroiditis). Together, these results further support adjusted CC screening strategies and targeted HPV vaccination in women with autoimmune conditions (41).

Our analyses are based on population-based biobank data, which offers access to large sample sizes, but at the same time, it can hinder the accessibility to more detailed clinical information (such as HPV status), especially when using summary-level data. Further studies evaluating the detected loci about specific HPV strains or histopathological features will elucidate their more specific role in cervical pathology etiopathogenesis. We used relatively simple phenotype definitions based solely on ICD codes, which on one hand simplifies data analysis, but on the other hand, may introduce unwanted heterogeneity as the use of these codes might somewhat vary in different healthcare systems. Additionally, since we used publicly available datasets, it was not possible to harmonise phenotype definitions and it cannot be ruled out that it may have some effect on the described results. However, we replicate many previously reported associations with CC, suggesting our approach is suitable. Although our study is the first attempt at a multi-ancestry GWAS meta-analysis, demonstrating similar effect estimates in both analysed ancestries, the number of non-European samples is small, and given the high prevalence of cervical malignancy in non-European populations, additional Black and Asian populations should be included in analyses to also improve the transferability of genetic risk scores.

In conclusion, our study provides the most comprehensive genetic analysis of cervical phenotypes to date. We characterise the genetics of benign cervical conditions ectropion and cervicitis, which is an important step toward a more complete understanding of cervical biology. We further clarify the genetic background of cervical malignancy, supporting the involvement of genes important for reproductive tract development, immune response, and cellular proliferation/apoptosis. The detailed characterisation of association signals, mapping of the causal variants and genes, and construction of the GRS make an important contribution to the scientific research of cervical biology and pathology. It allows for classifying disease sub-phenotypes or running follow-up phenome-wide association studies but also offers necessary background knowledge to further functional studies, which may pave the way to better treatment and prevention of cervical neoplasia.

## Materials and Methods

### Study design and participants

#### Estonian biobank

The EstBB is a population-based biobank with genotype data and health information for over 200 000 participants (42). Information on International Classification of Disease-10 (ICD10) codes is obtained via regular linking with the Health Insurance Fund and other relevant registries (43). The 150 K data freeze was used for the genetic association analyses described in this paper (n = 92 042 women). All biobank participants have signed a broad informed consent for using their data in research and the study was carried out under ethical approval 1.1-12/624 from the Estonian Committee on Bioethics and Human Research (Estonian Ministry of Social Affairs) and data release N05 from the EstBB.

Using individual-level data, ICD10 codes N86 (Erosion and ectropion of cervix uteri), N72 (Inflammatory disease of cervix uteri), N87 (Dysplasia of cervix uteri) and C53/D06 (Cervical cancer) were used for extracting cases. Women who did not have the respective ICD codes were used as controls. The final sample size included for analysis was as follows: cervical ectropion: 9664 cases (average age at joining the biobank $\pm$ SD, 35.7 $\pm$ 9.6 years), 82 378 controls (45.1 $\pm$16.3); cervicitis: 18 192 cases (40.9 $\pm$ 11.9), 73 850 controls (44.9 $\pm$ 16.8); cervical dysplasia: 10 448 cases (39.6 $\pm$ 12.1), 81 594 controls (44.6 $\pm$ 16.4); CC 748 cases (50.3 $\pm$ 13.4), 81 870 controls (44.6 $\pm$ 16.3) (Supplementary Material, Table S11). The overlap of cases for each phenotype can be seen in Supplementary Material, Fig. S10. For follow-up analyses, we further stratified the dysplasia cases by severity, resulting in 4250 cases with mild (N87.0), 2616 with moderate (N87.1) and 1599 with severe dysplasia, not elsewhere classified (N87.2), respectively. If more than one diagnosis code was present for dysplasia/cancer, we selected the most severe for analysis (mild < moderate < severe dysplasia < CC). To validate the cancer diagnosis in the EstBB, we compared the diagnoses for cases (obtained via linking with the National Health Insurance Fund and from self-reported data) to those available through the Estonian Cancer Registry. Reporting cancer cases to the Cancer Registry is compulsory for all physicians in Estonia who diagnose or treat cancer. Data are also submitted by forensic pathologists. When comparing the same period (diagnoses up to 2016-12-29), out of 707 individuals with C53/D06 diagnosis from other sources, 69% also had the C53/D06 diagnosis in the Cancer Registry. It should be noted that the most recent linking with Cancer Registry includes data up to the end of 2016, therefore, the actual overlap is likely higher, as linking with the National Health Insurance Fund has also been done periodically after this date and, for a subset of the individuals, the diagnosis is not yet reflected in the Cancer Registry Data.

All EstBB participants have been genotyped at the Genotyping Core Lab of the Institute of Genomics, University of Tartu, using Illumina Global Screening Array v1.0 and v2.0. Samples were genotyped and PLINK format files were created using Illumina GenomeStudio v2.0.4. Individuals were excluded from the analysis if their call rate was < 95% or if the sex defined based on heterozygosity of the X chromosome did not match the sex in phenotype data. Before imputation, variants were filtered by call rate < 95%, HWE *P*-value <1e−4 (autosomal variants only) and minor allele frequency < 1%. Human genome b37 was used and all variants were changed to be top strand of DNA (TOP) strand using GSAMD-24v1-0_20011747_A1-b37.strand.RefAlt.zip files from https://www.well.ox.ac.uk/~wrayner/strand/webpage. Prephasing was done using Eagle v2.3 software (44) (the number of conditioning haplotypes Eagle2 uses when phasing each sample was set to: Kpbwt= 20 000) and imputation was done using Beagle v.28Sep18.79339 with effective population size *n*_e_ = 20 000. Population-specific imputation reference of 2297 whole genome sequencing (WGS) samples was used (45). Association analysis was carried out using SAIGE software implementing a mixed logistic regression model (46), using the year of birth and 10 PCs as covariates in step I.

#### FinnGen study

The FinnGen study is a public-private partnership bringing together genotyping data from different Finnish Biobanks and electronic health records from Finnish health registries. FinnGen release 5 (R5) data, consisting of 218 792 individuals were used, and summary statistics for the following pre-defined phenotypes of interest defined using ICD10, ICD9 and ICD8 codes were used: cervical ectropion (n_cases_ = 498, n_controls_ = 68 969), cervicitis (n_cases_ = 1093, n_controls_ = 111 858) and cervical dysplasia (n_cases_ = 4246, n_controls_ = 68 969). Additionally, publicly available GWAS summary statistics for phenotype ‘Malignant neoplasm of cervix uteri’ from freeze R4 (n_cases_ = 1313, n_controls_ = 99 048) was used. Since we had only access to summary-level data, we have no information on the descriptive statistics (age range) of the sample. In short, phenotypes were defined as follows: ectropion (N14_EROSECTROPUT, ICD10 N86, ICD9 6220A, ICD8 62 191) and ectropion controls excluding non-inflammatory diseases of the female genital tract (N80–N98), cervicitis (N14_INFCERVIX, ICD10 N72, ICD9 616 and ICD 620) and cervicitis controles excluding inflammatory diseases of the female genital tract (N70-N77), dysplasia (N14_DYSPLACERVUT ICD10 N87, ICD9 6221 and ICD8 621) and dysplasia controls—excluding non-inflammatory diseases of the female genital tract (N80-N98), cervical cancer (C3_CERVIX_UTERI, ICD10 C53, ICD9 180 and ICD8 180) and cervical cancer are individuals that are not cases. More detailed information on FinnGen endpoint definitions can be found at https://www.finngen.fi/en/researchers/clinical-endpoints. FinnGen individuals were genotyped with Illumina and Thermo Fisher arrays and imputed to the population-specific SISu v3 imputation reference panel according to the following protocol: dx.doi.org/10.17504/protocols.io.xbgfijw. Genetic association testing was carried out with SAIGE (46). FinnGen summary statistics included prefiltered variants (minimum allele count>5, imputation INFO score > 0.6) and variant positions were converted to b37 using the binary liftOver tool (https://genome.sph.umich.edu/wiki/LiftOver#Binary_liftOver_tool). For more information on genotype data, disease endpoints and GWAS analyses, please see https://finngen.gitbook.io/documentation/. Patients and control subjects in FinnGen provided informed consent for biobank research, based on the Finnish Biobank Act. Alternatively, separate research cohorts, collected before the Finnish Biobank Act came into effect (in September 2013) and the start of FinnGen (August 2017), were collected based on study-specific consents, and later transferred to the Finnish biobanks after approval by Fimea, the National Supervisory Authority for Welfare and Health. Recruitment protocols followed the biobank protocols approved by Fimea. The Coordinating Ethics Committee of the Hospital District of Helsinki and Uusimaa (HUS) approved the FinnGen study protocol Nr HUS/990/2017.

The FinnGen study is approved by Finnish Institute for Health and Welfare (THL), approval number THL/2031/6.02.00/2017, amendments THL/1101/5.05.00/2017, THL/341/6.02.00/2018, THL/2222/6.02.00/2018, THL/283/6.02.00/2019, THL/1721/5.05.00/2019, digital and population data service agency VRK43431/2017–3, VRK/6909/2018–3, VRK/4415/2019–3 the Social Insurance Institution (KELA) KELA 58/522/2017, KELA 131/522/2018, KELA 70/522/2019, KELA 98/522/2019 and Statistics Finland TK-53-1041-17.

The Biobank Access Decisions for FinnGen samples and data utilised in FinnGen Data Freeze 5 include THL Biobank BB2017_55, BB2017_111, BB2018_19, BB_2018_34, BB_2018_67, BB2018_71, BB2019_7, BB2019_8, BB2019_26, Finnish Red Cross Blood Service Biobank 7.12.2017, Helsinki Biobank HUS/359/2017, Auria Biobank AB17–5154, Biobank Borealis of Northern Finland_2017_1013, Biobank of Eastern Finland 1186/2018, Finnish Clinical Biobank Tampere MH0004, Central Finland Biobank 1–2017 and Terveystalo Biobank STB 2018001.

*Publicly available datasets:* For CC meta-analysis we additionally used publicly available datasets from Rashkin *et al.* 2020 (4) (downloaded from https://github.com/Wittelab/pancancer_pleiotropy, including 5998 cases and 189 855 controls from the UK Biobank, and 565 cases and 29 801 controls from Kaiser Permanente cohort) and summary statistics from Biobank Japan (47) (downloaded from http://jenger.riken.jp/en/result), including 605 cases and 89 731 controls. The summary statistics for Biobank Japan included variant-level association statistics needed for the meta-analysis (effect estimate the beta, standard error (SE), effect allele, another allele, sample size and effect allele frequency, association *P*-value, etc.). The summary statistics from Rashkin *et al.* study contained OR and *P*-values, therefore, to use this cohort in the meta-analysis, we first converted OR-s to betas [beta = log(OR)], then derived z-scores from reported *P*-values (using the ‘qnorm’ function in R) and calculated SE-s (SE = beta/z-score).

*GWAS meta-analysis:* All EUR meta-analyses were conducted using inverse variance weighted fixed-effect meta-analysis method implemented into GWAMA software (v2.2.2) (48). For CC meta-analysis including data from Biobank Japan, we used MR-MEGA, which is a tool for multi-ancestry meta-regression (49). Genome-wide significance was set at *P* < 5 $\times$ 10^−8^ in all analyses. We used MTAG v1.0.8 (50) (Multi-Trait Analysis of GWAS) to jointly analyse the summary statistics from dysplasia and CC EUR analyses and thus increase the power to detect additional associations.

Variant annotation and follow-up analyses were done using individual trait GWAS summary statistics from EUR analyses.

Summary-level meta-analysis statistics can be accessed from the GWAS catalogue (GCST90246355, GCST90246356, GCST90246357, GCST90246358 and GCST90246359).

*Annotation of GWAS signals:* We used Functional Mapping and Annotation of Genome-Wide Association Studies (FUMA) v.1.3.6 (51) for functional annotation of GWAS results and credible set variants. For functional annotation, the annotate variation (52), Combined Annotation Dependent Depletion (CADD), (a continuous score showing how deleterious the SNP is to protein structure/function, where; scores > 12.37 indicate potential pathogenicity) (53) and RegulomeDB (54) scores (ranging from 1 to 7, where a lower score indicates greater evidence for having regulatory function), as well as 15 chromatin states from the Roadmap Epigenomics Project (55) were used. FUMA also performs lookups in the GWAS catalogue (e96_r2019-09-24), the results of which are shown in Supplementary Material, Table S4 and Supplementary Material, Fig. S5.

*Look-up of variants previously associated with cervical cancer:* We used our EUR only and multi-ancestry CC meta-analysis summary statistics to conduct a look-up of variants previously reported in association with cervical carcinoma. For this, we extracted variants associated with the Experimental Factor Ontology (EFO) term EFO_0001061(cervical carcinoma) from the GWAS catalogue. The results of this look-up can be seen in Supplementary Material, Table S9.

*Gene-based tests:* We used MAGMA (v1.08) (56) implemented in FUMA with default settings to conduct gene-based genome-wide association testing. According to the number of tested protein-coding genes, the genome-wide significance level was set at 0.05/19913 = 2.7 × 10^−6^

*HLA analysis:* For cervical dysplasia meta-analysis, we carried out HLA imputation of the EstBB genotype data with the SNP2HLA v1.0.3 tool (57). As an imputation reference, we used a merged reference of EstBB WGS (45) and Type 1 Diabetes Genetics Consortium samples (57). We tested for association between the alleles and cervical dysplasia in the EstBB using SAIGE with the LOCO option. We used imputed data on alleles (two- and four-digit) in the MHC class I genes (*HLA-A*, *HLA-B* and *HLA-C*) and classical MHC class II genes (*HLA-DRB1*, *HLA-DQA1*, *HLA-DQB1*, *HLA-DPA1* and *HLA-DPB1*) for 10 446 cases and 81 586 controls in the EstBB, who had the corresponding data available.

*Colocalisation and fine-mapping analyses:* We used HyPrColoc (v1.0.0) (58), a fast and efficient colocalisation method for identifying the overlap between our GWAS meta-analysis signals and *cis*-QTL signals from different tissues and cell types (expression QTLs, transcript QTLs, exon QTLs and exon usage QTLs available in the eQTL catalogue) (59). We lifted the GWAS summary statistics over to the hg38 build to match the eQTL catalogue using the binary liftOver tool (https://genome.sph.umich.edu/wiki/LiftOver#Binary_liftOver_tool). For each genome-wide significant (*P* < 5 $\times$ 10^−8^) GWAS locus we extracted the +/−500 kb of its top hit from QTL datasets and ran the colocalisation analysis against eQTL catalogue traits. For each eQTL catalogue dataset, we included all the QTL features which shared at least 80% of tested variants with the variants present in our GWAS region. We used the default settings for HyPrColoc analyses and did not specify any sample overlap argument, because the HyPrColoc paper (58) demonstrates that assuming trait independence gives reasonable results. HyPrColoc outputs the following results (a) a cluster of putatively colocalised traits—(here our GWAS region of interest and *cis*-QTL signal for any nearby feature for a given QTL dataset); (b) the PP that genetic association signals for those traits are colocalising—(we considered two or more signals to colocalise if the PP for a shared causal variant (PP4) was 0.8 or higher. All results with a PP4 > 0.8 can be found in Supplementary Material, Table S3); (c) the ‘regional association’ probability—(a large regional association probability indicates that one or more SNPs in the region have shared association across evaluated traits); (d) a candidate causal variant explaining the shared association; and (e) the proportion of the PP explained by this variant—(which also represents the HyPrColoc multi-trait fine-mapping probability). For every colocalisation event, we also calculated a 95% credible set for multi-trait fine-mapping results. To do so, we ranked all variants decreasingly based on their PP and extracted top *n* variants with a cumulative PP of ≥0.95.

Since cervical samples were not present in analysed gene expression datasets, we prioritised colocalisation signals from tissues that cluster together with vagina/uterus in GTEx V8 data, either based on cell-type-composition or gene expression [Supplementary Material, Figs S41 and S48 of (60)]. These tissues include the vagina, uterus, oesophagus mucosa and gastro-oesophageal junction, sigmoid colon, skin, salivary gland and tibial nerve. Of these ‘proxy’ tissues, oesophageal mucosa—(stratified squamous epithelium) and gastro-oesophageal junction—(transition zone between stratified and columnar epithelium), tissues are histologically most similar to the cervix.

We used FUMA (51) to annotate credible set variants with chromatin 15-state marks in the HeLa-S3 Cervical Carcinoma cell line (E117) and in available ‘proxy’ tissues (E106—a sigmoid colon; E079—oesophagus; E055-E061, E126, E127—skin) from the Roadmap Epigenomics Project (55).

*Genetic correlations:* We used the LD Score regression (LDSC) method (61) implemented in LD Hub (28) (http://ldsc.broadinstitute.org) for testing genetic correlations between CC and traits spanning reproductive, aging, autoimmune, cancer and smoking behaviour categories (33 traits in total), using the CC European-ancestry only GWAS meta-analysis summary statistics and data available within the LD Hub resource. After filtering the input to HapMap3 SNPs, removing SNPs within the HLA region, and merging with the built-in reference panel LD Scores (1000 Genomes EUR ancestry) (29), ~ 1.1 M variants remained for analysis. FDR correction (calculated using the p.adjust function in R) was used to account for multiple testing. The results of the analysis are presented in Supplementary Material, Table S10.

LDSC-estimated observed scale heritability [0.0059 (se = 0.0013)] for CC was converted to liability scale using the formula h^2^_liability_ = h^2^_observed_  $\times$K^2^  $\times$ (1—K)^2^/P/(1−P)/zv^2^, where K is the population prevalence (here equal to sample prevalence) and P is the proportion of cases in the study (EUR analysis, 2.1%). This resulted in a liability scale heritability estimate of 4.75% for non-HLA common variant heritability.

*Genetic risk score analysis:* We constructed a GRS for CC based on the summary statistics of the meta-analysis including the data from Rashkin *et al.* and FinnGen, with 7876 cases and 318 704 controls of EUR, leaving out EstBB 200 K dataset as an independent target dataset (1094 CC cases and 131 314 female controls) (Supplementary Material, Fig. S11).

We computed and evaluated 10 versions of GRS for each individual in the EstBB 200 K dataset (132 408 women, 70 502 men) implementing LDPred (62), which uses a linkage-disequilibrium SNP-reweighting approach. The following fractions of causal variants were used: 1, 0.3, 0.1, 0.03, 0.01, 0.003, 0.001, 0.0003 and 0.0001. LD data from the Estonian WGS dataset (n = 2297) was used for reference. STEROID (v0.1.1) was used for calculating GRS for all EstBB participants (https://genomics.ut.ee/en/tools/steroid).

First, we divided the target EstBB dataset into a discovery (prevalent cases) and validation (incident cases) dataset. The discovery dataset included 859 prevalent cases and 3436 controls (four controls per case). Since controls were defined as women who did not develop CC during follow-up, they tended to be younger than prevalent cases. We used the discovery set to select the best predicting GRS version using a logistic regression model adjusted for age, age squared and smoking status (coded as ‘Never’, ‘Former’ and ‘Current’). We used smoking status as a covariate, as it is a known risk factor for CC and was easily available for all included biobank participants. The GRS that had the largest AUC and smallest *P*-value in the discovery set analysis (2 894 555 variants and causal fraction 0.003), was selected for further analyses. This GRS can be accessed from the PGS Catalog (ID: PGS003428).

The validation set included 235 incident cases and 127 878 controls, and in this set, we tested the predictive ability of GRS (Supplementary Material, Fig. S11). We standardised the best GRS version and also categorised it into different percentiles (<5%, 5–15%, 15–25%, 25–50%, 50–75%, 75–85%, 85–95%, > 95%). Cox proportional hazard models were used to estimate the HR corresponding to one SD of the continuous GRS for the validation dataset. Harrell’s C-statistic was used to characterise the discriminative ability of each GRS. Cumulative incidence estimates were computed using Kaplan–Meier method and to account for competing events (mortality), we used the ‘cmprsk’ R library. While comparing different GRS groups with each other, age was used as a timescale to properly account for left truncation in the data.

To explore how much of the GRS predictive power comes from the HLA region, we separated the GRS into HLA and non-HLA fractions. We took the best-performing GRS and extracted the markers and LDpred weights in the HLA region (chr6:28477797-33448354, number of variants = 9764) and calculated separate scores for the HLA region using STEROID. We then subtracted the HLA score from the overall score, resulting in a non-HLA score.

We also performed a pheWAS analysis with the best-performing GRS, where we tested the association between the GRS and all ICD10 diagnosis codes in EstBB 200 K data (excluding relatives using a pi-hat cut-off value 0.2) in a logistic regression framework, adjusting for sex, age and 10 PCs. Separate analyses stratified by sex were also performed. Bonferroni correction was applied to select statistically significant associations (number of tested ICD main codes—2001, corrected *P*-value threshold 0.05/2001 = 2.5 $\times$ 10^−5^.). We repeated the overall pheWAS analysis with the HLA and non-HLA scores to clarify which fraction of the score drove the associations. Results were visualised using the PheWas library (https://github.com/PheWAS/PheWAS). All analyses were carried out in R 3.6.1 or R 4.1.1.

## Supplementary Material

Supplementary_Figures_ddad043

Cervical_phenotypes_manuscript_supplementary_tables_010222_ddad043
