# Supplementary material for: GWAS meta-analyses clarify the genetics of cervical phenotypes and inform risk stratification for cervical cancer
Source: Hum Mol Genet. 2023 Mar 16;32(12):2103–16. doi: 10.1093/hmg/ddad043 (PMC10244231; doi:10.1093/hmg/ddad043)
Supplement: Supplementary_Figures_ddad043 [file supplementary_figures_ddad043.zip › Supplementary_Figures_ddad043.docx]

**Supplementary**

| A  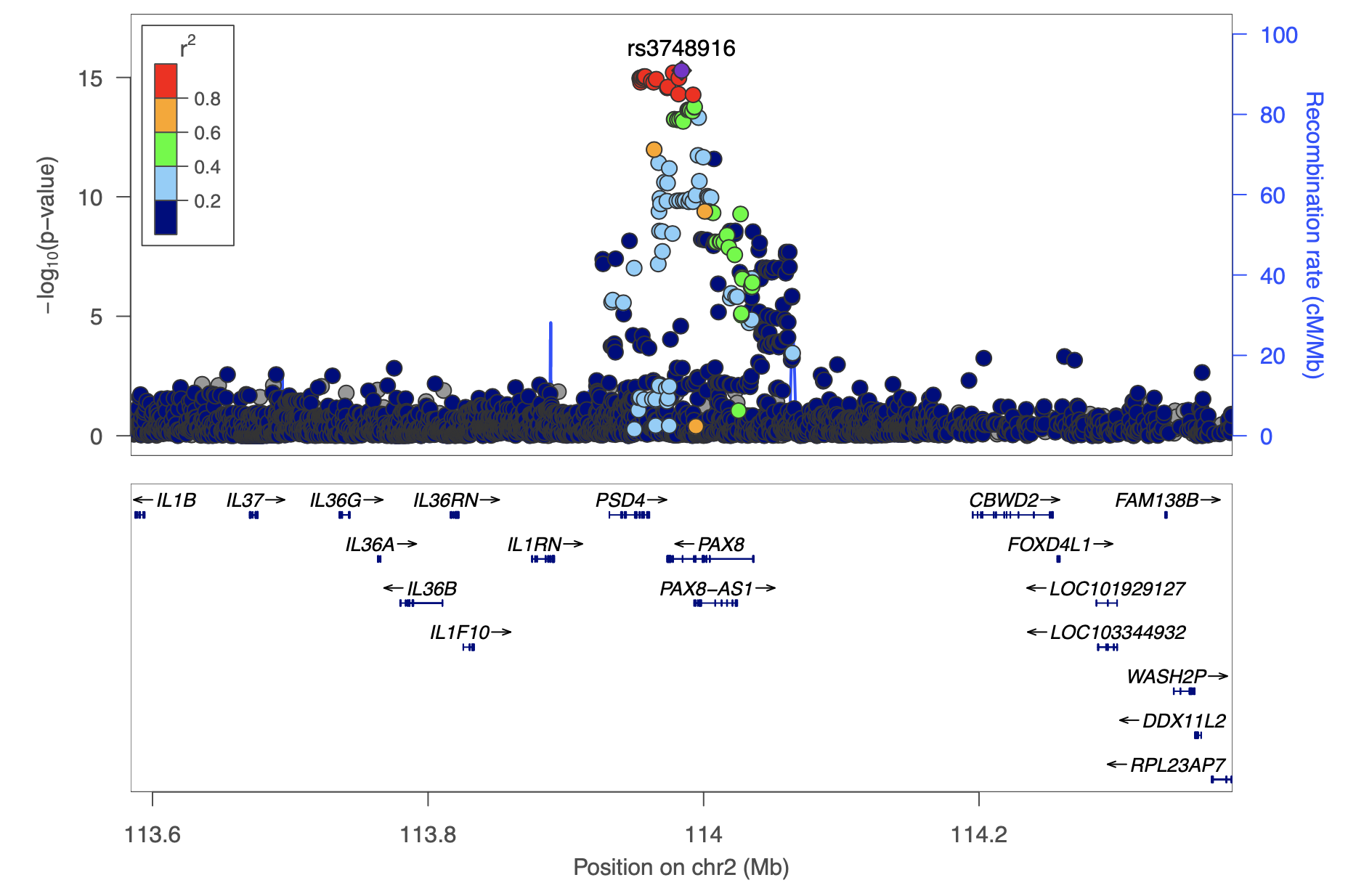 | B  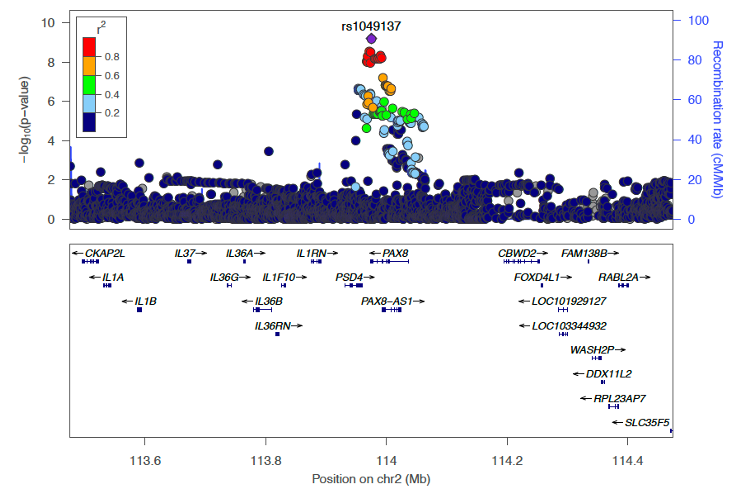 |
| --- | --- |

**Supplementary Figure 1.** Regional plots for cervical ectropion (A) and cervicitis (B) GWAS meta-analysis signals on chromosome 2.

| A  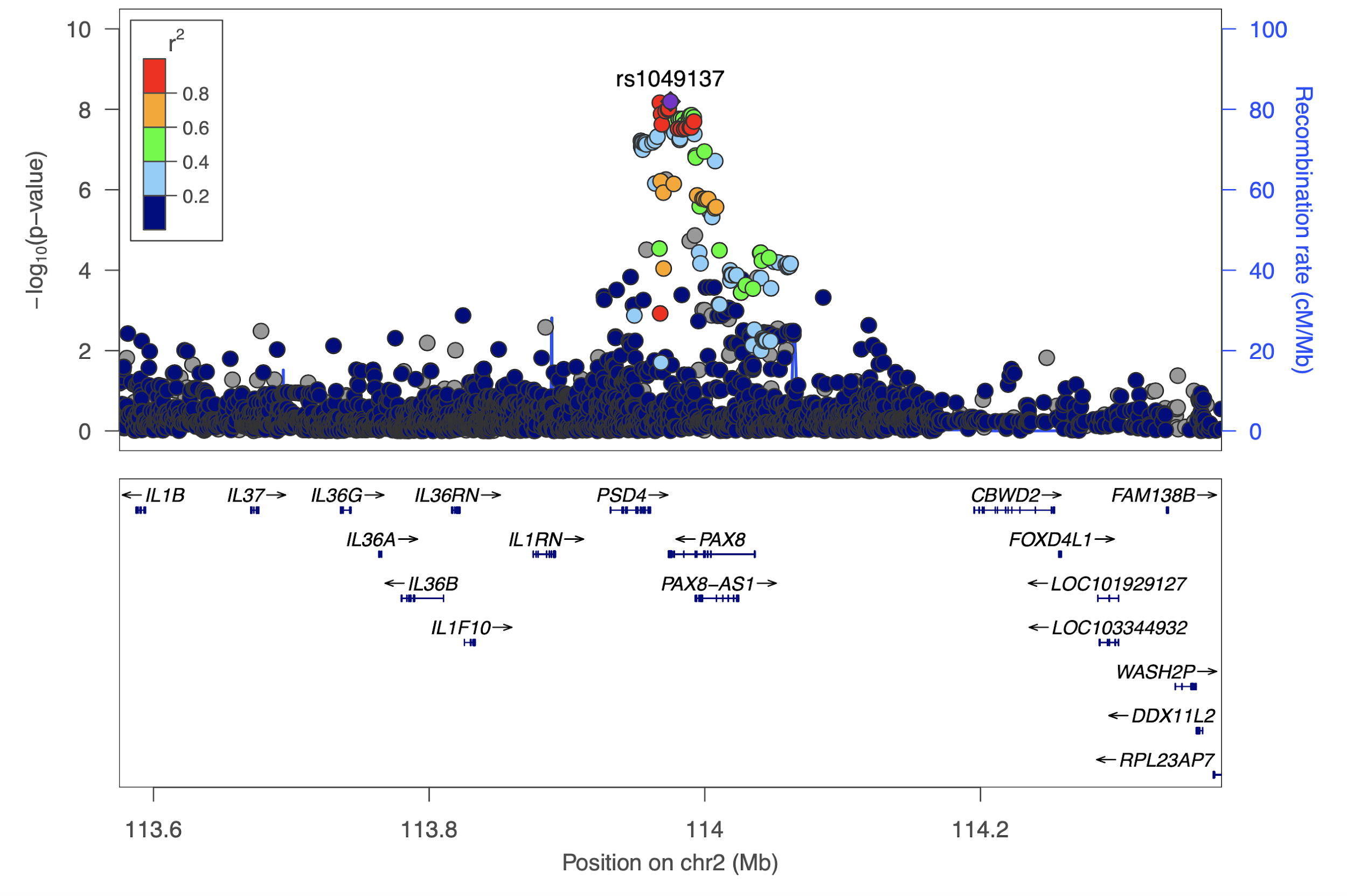 | B  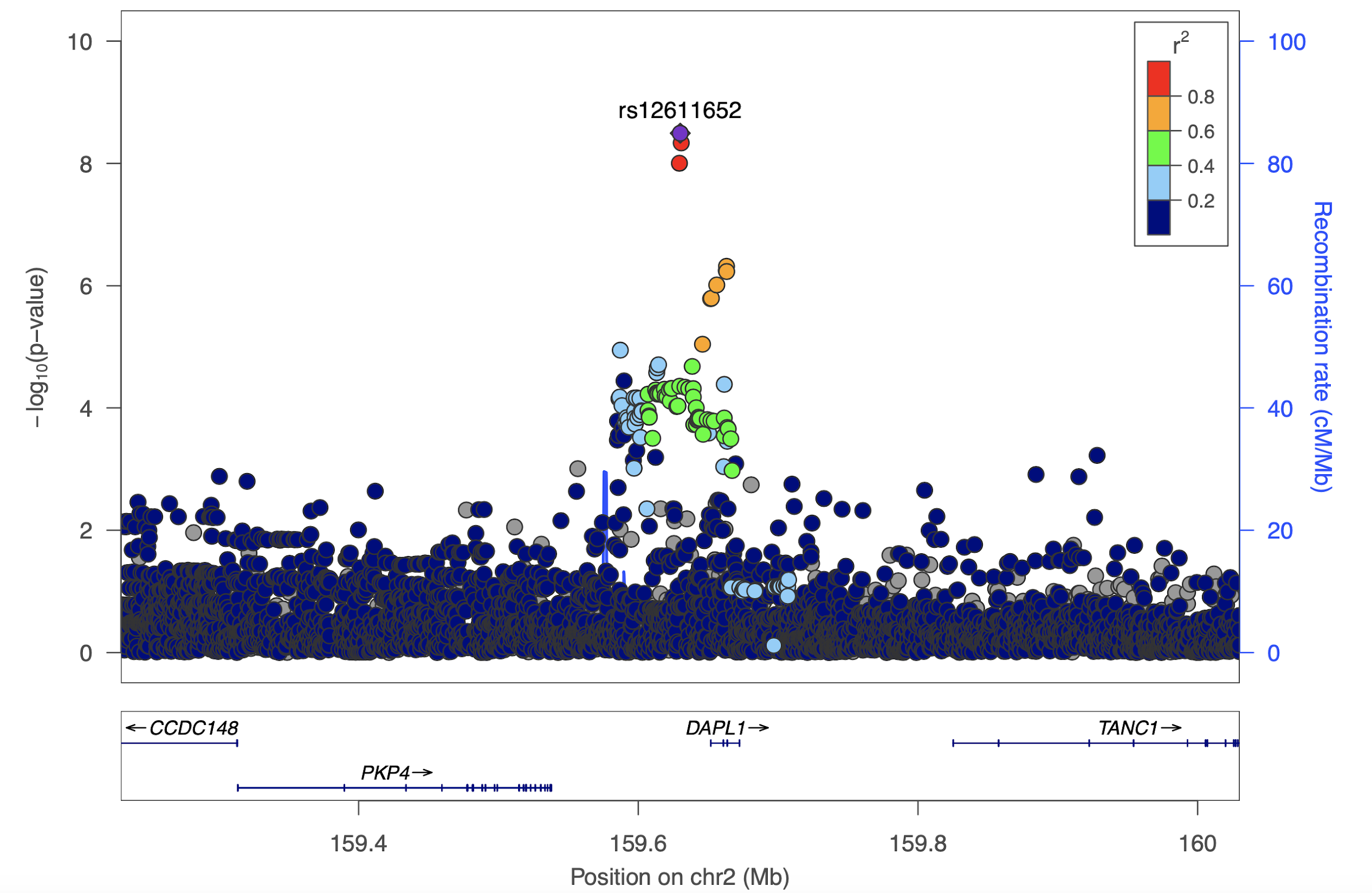 | C  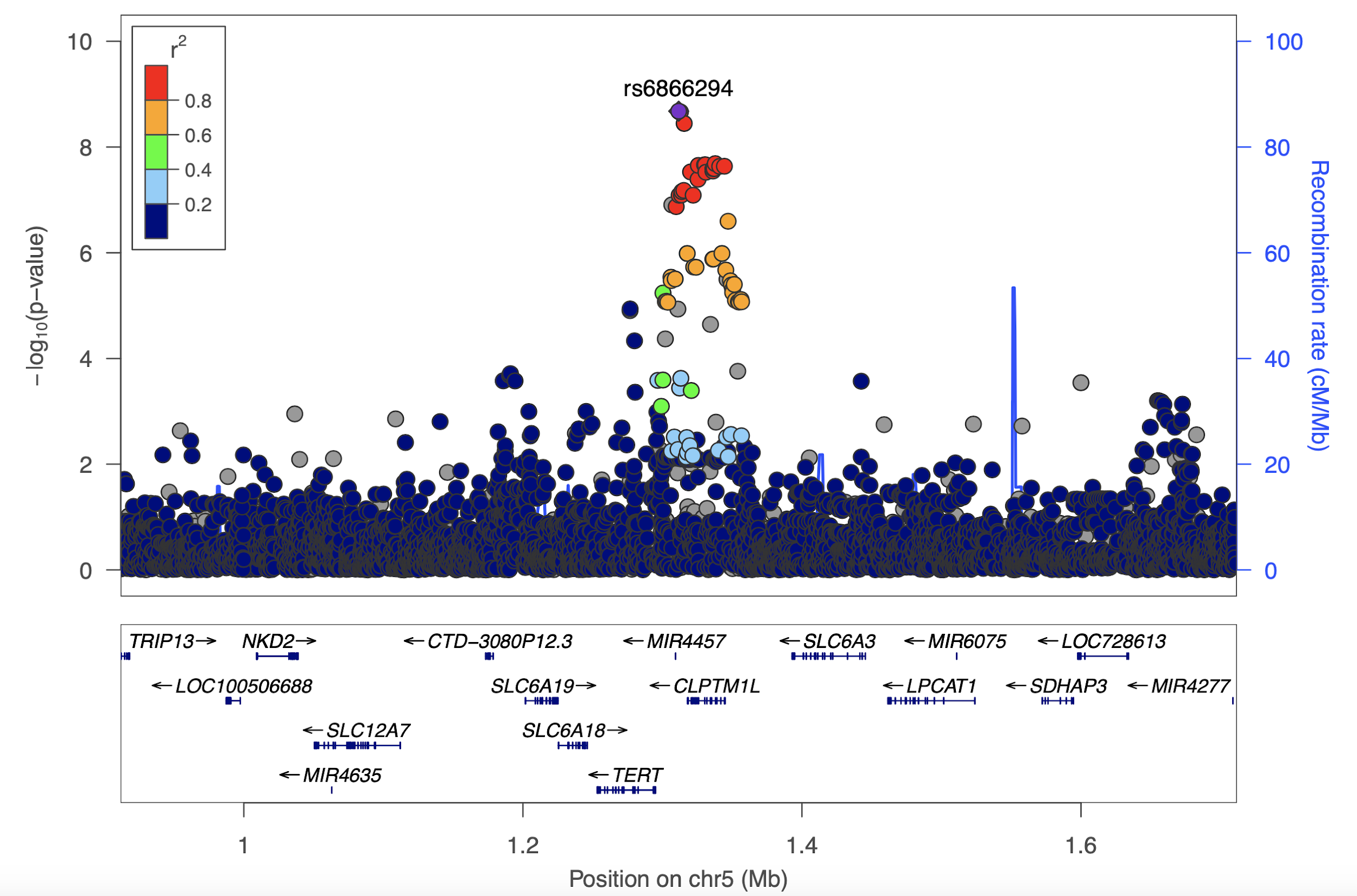 |
| --- | --- | --- |
| D  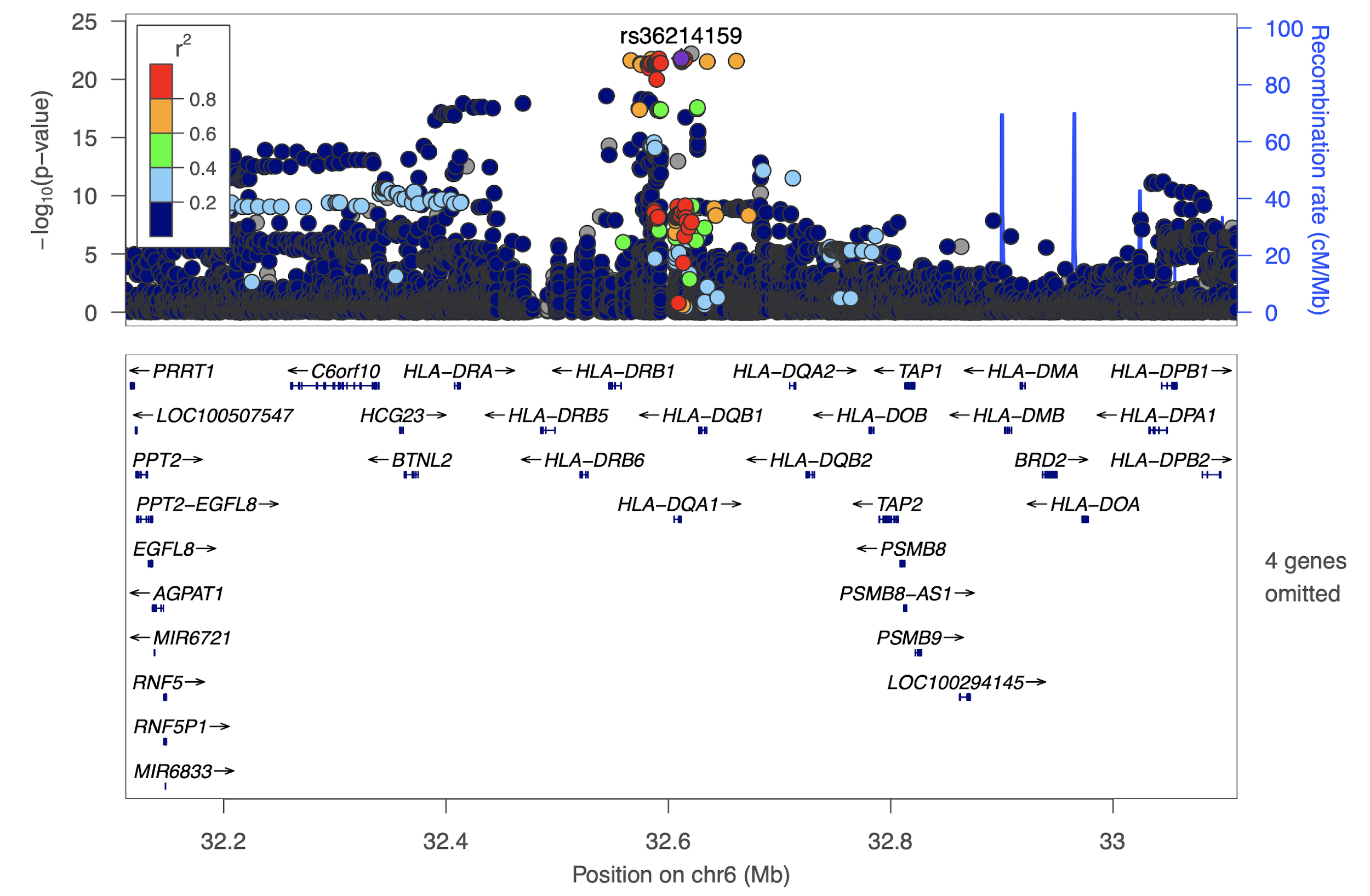 | E  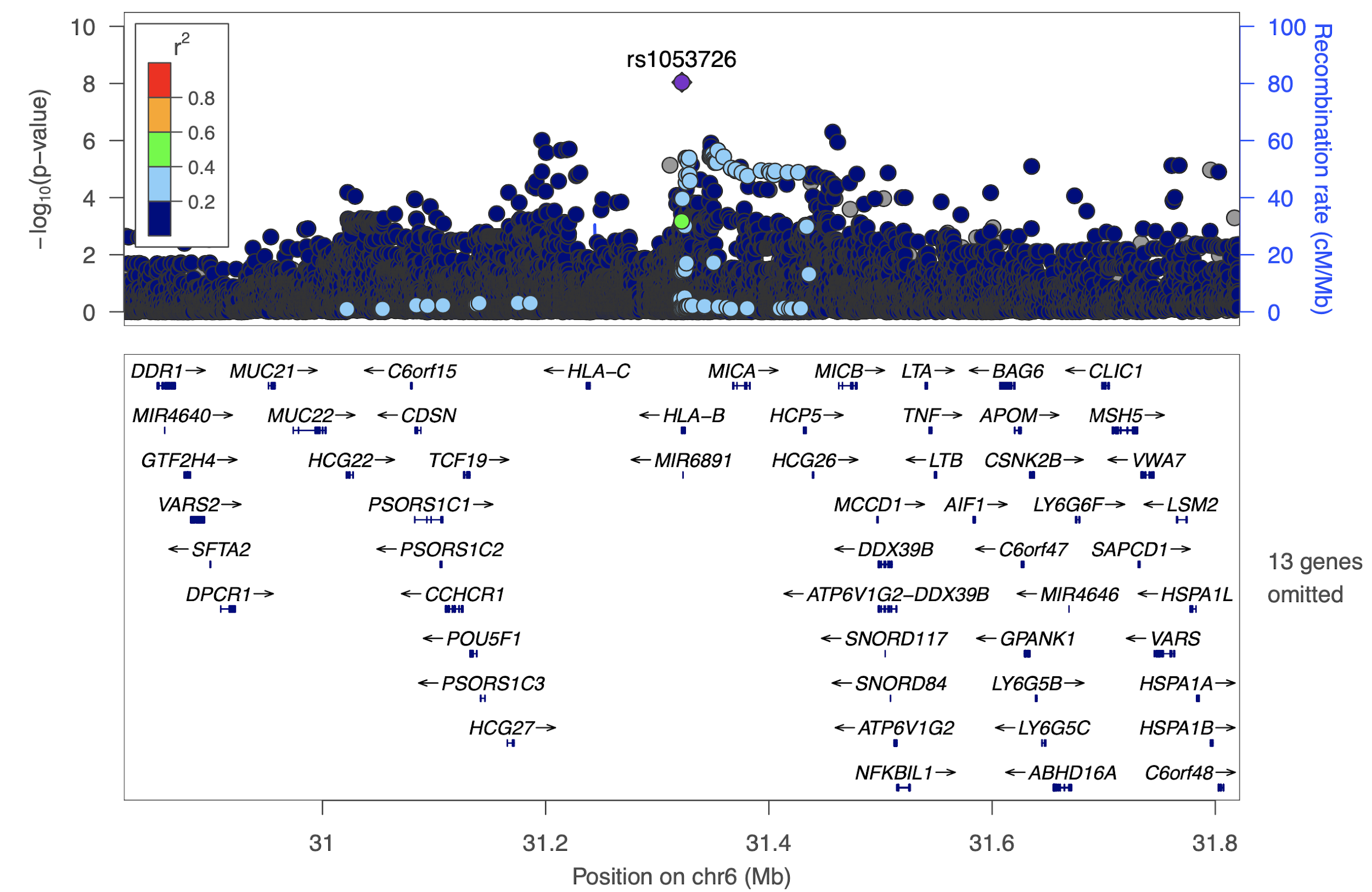 | **Supplementary Figure 2.** Regional plots for cervical dysplasia GWAS meta-analysis signals on chr2 (A, B), chr5 (C) and chr6 (D, E). |

| A  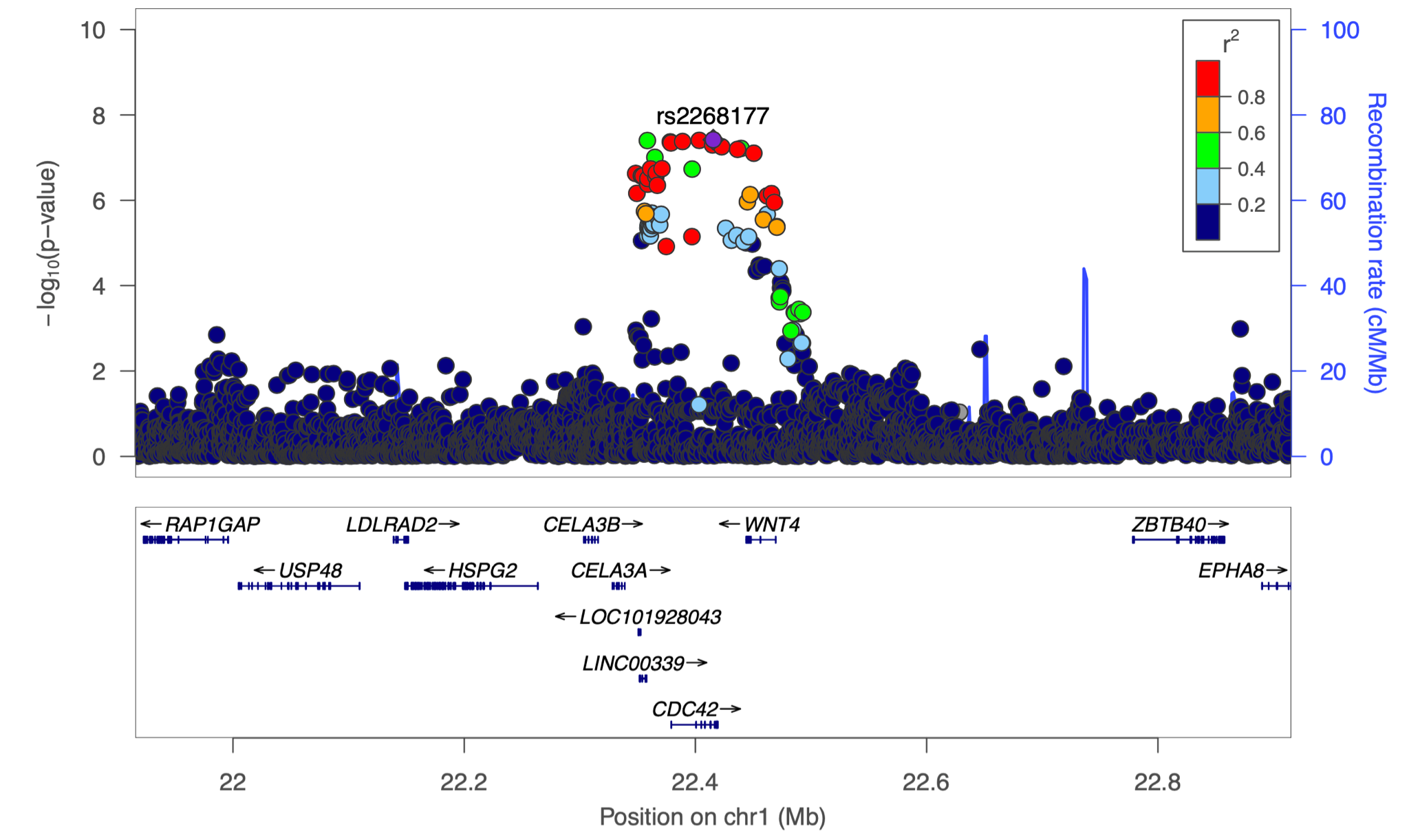 | B  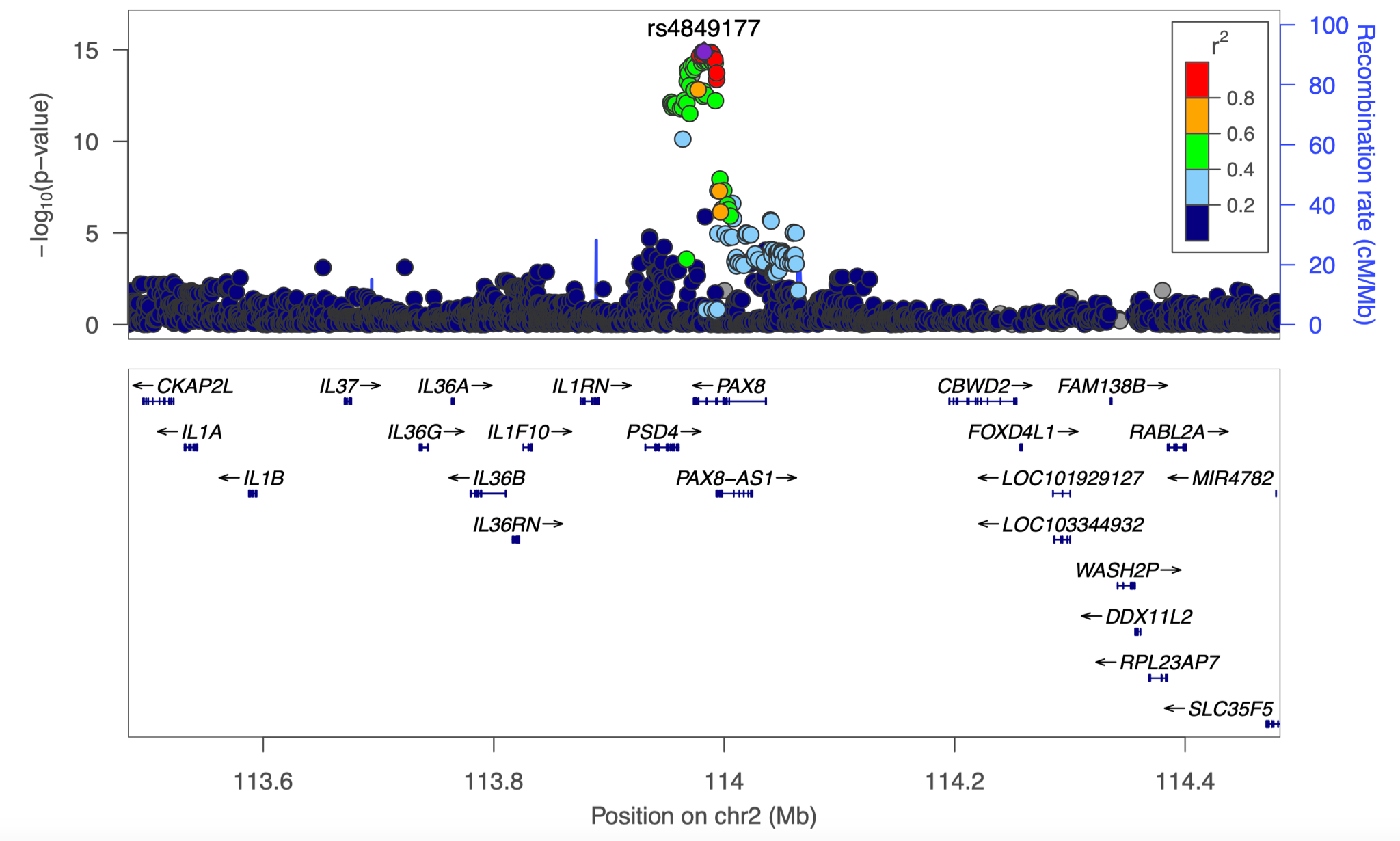 | C  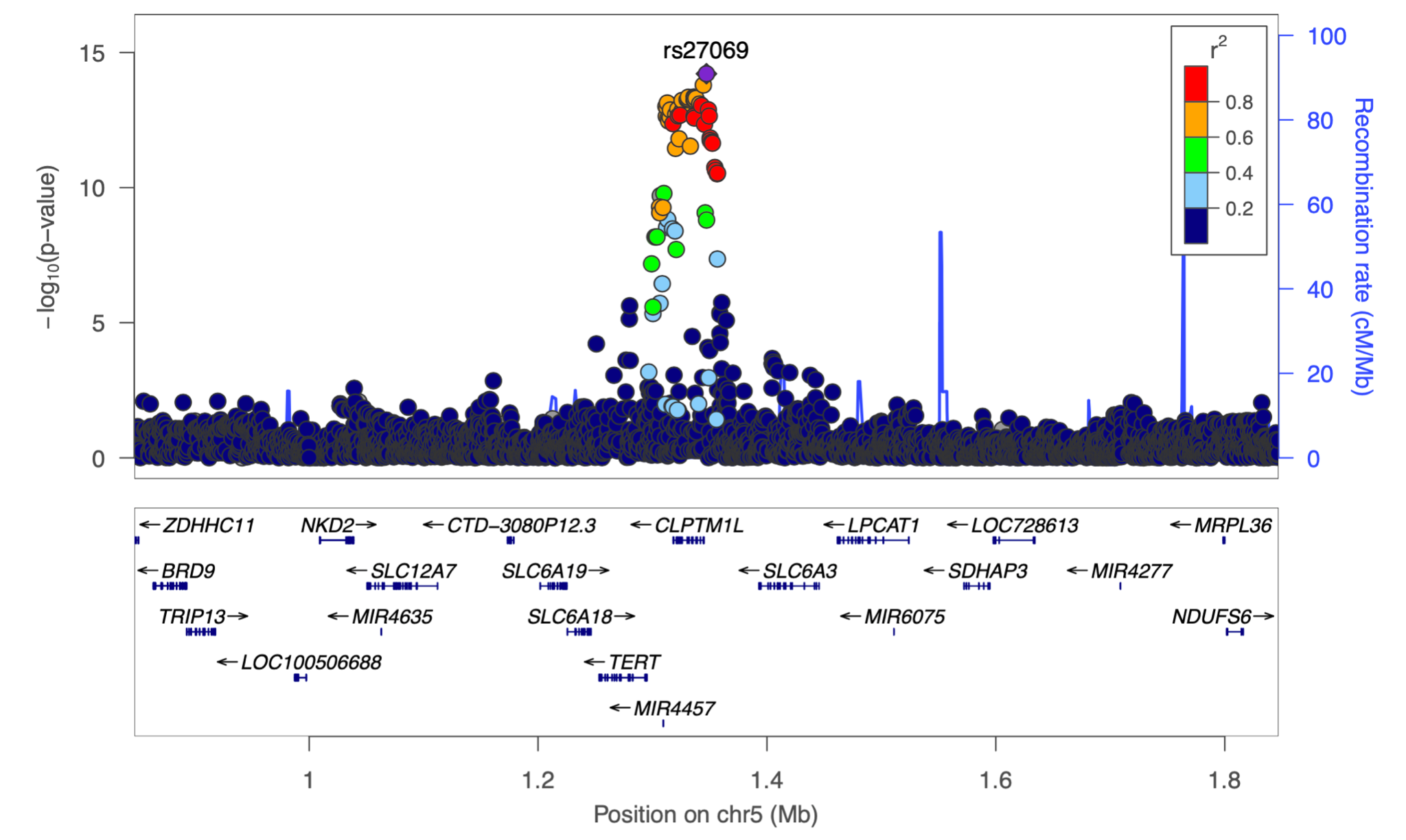 |
| --- | --- | --- |
| D  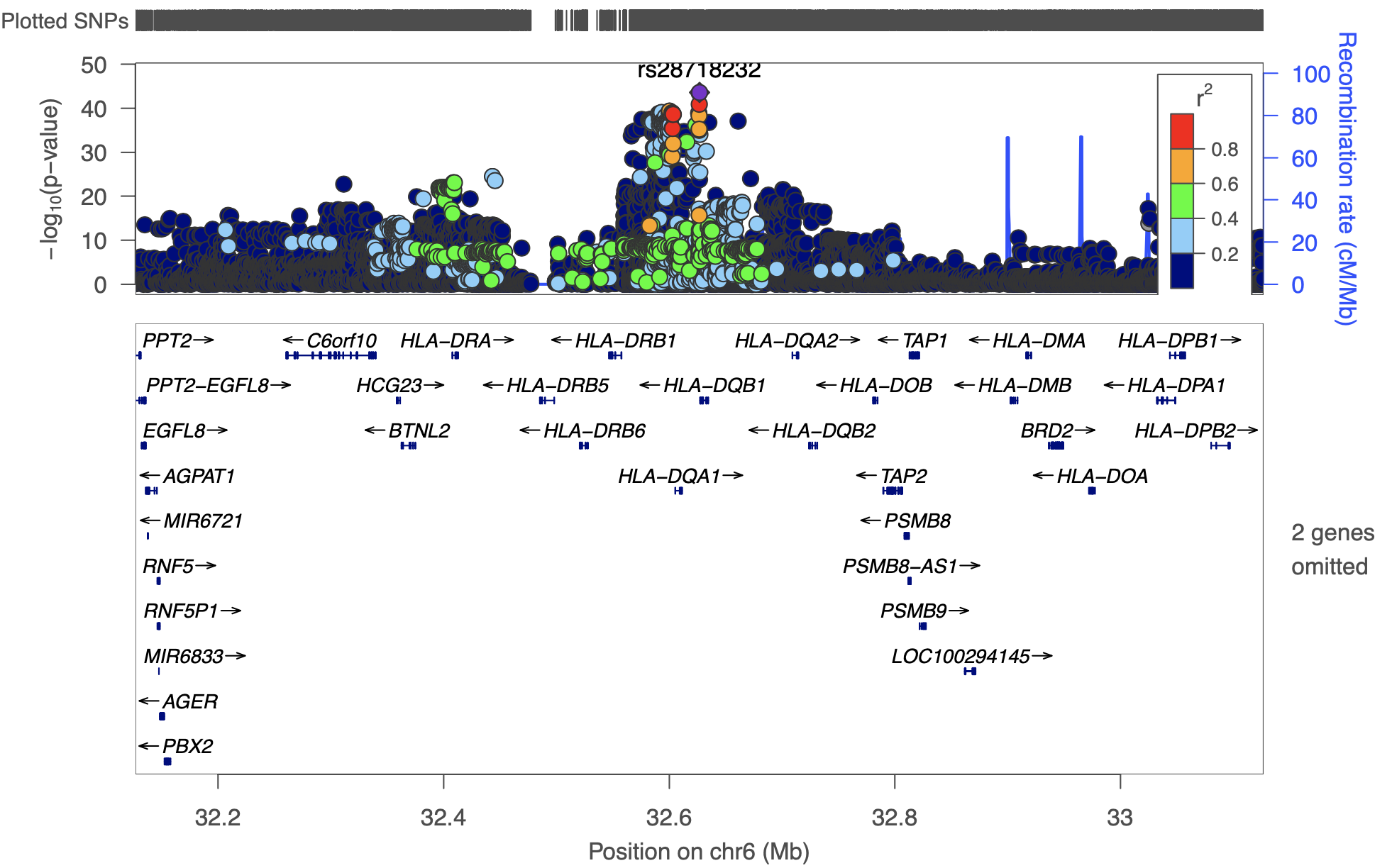 | E  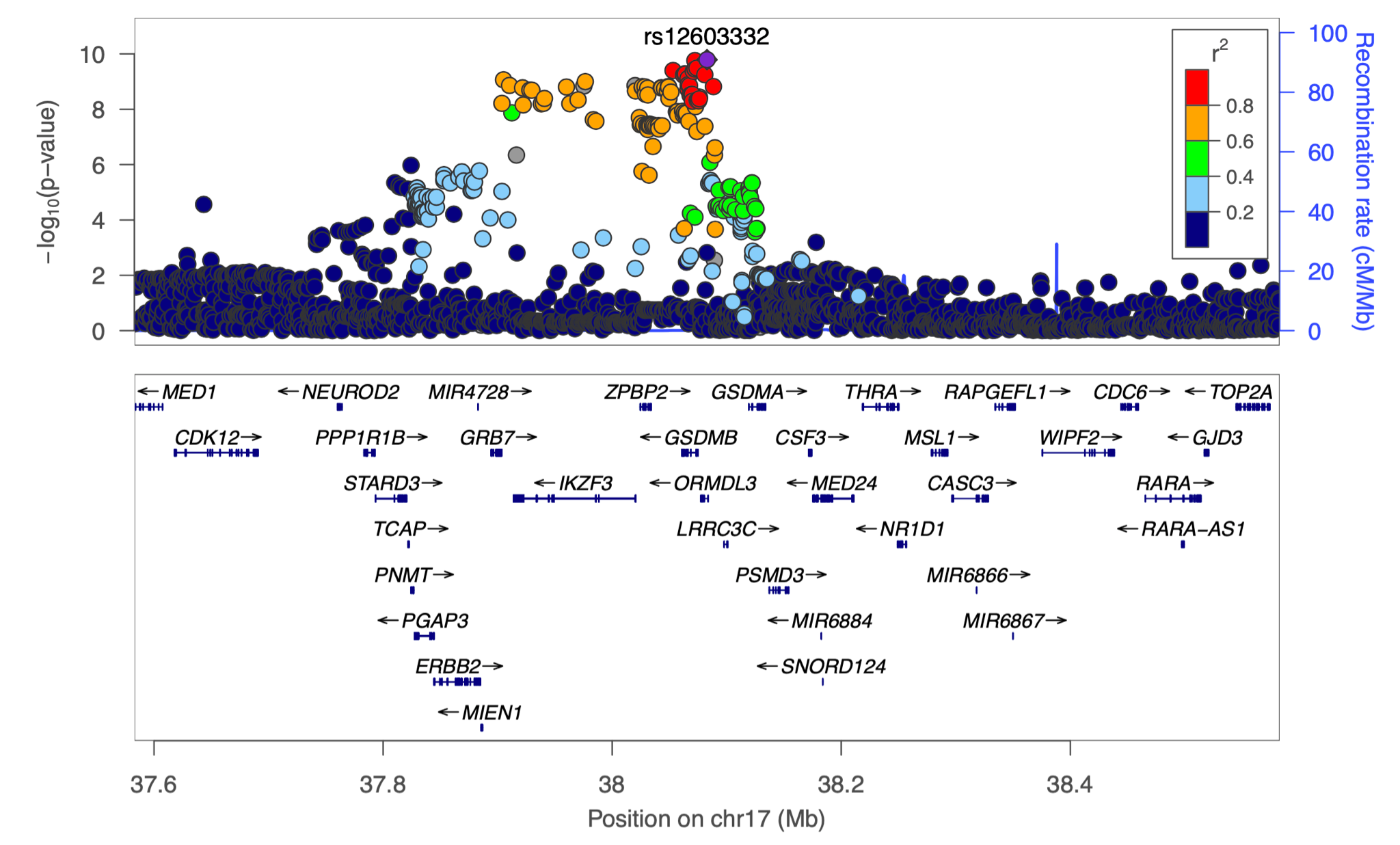 | **Supplementary Figure 3.** Regional plots for cervical cancer European ancestry GWAS meta-analysis signals on chr1 (A), chr2 (B), chr5 (C), chr6 (D) and chr17 (E). |

| A  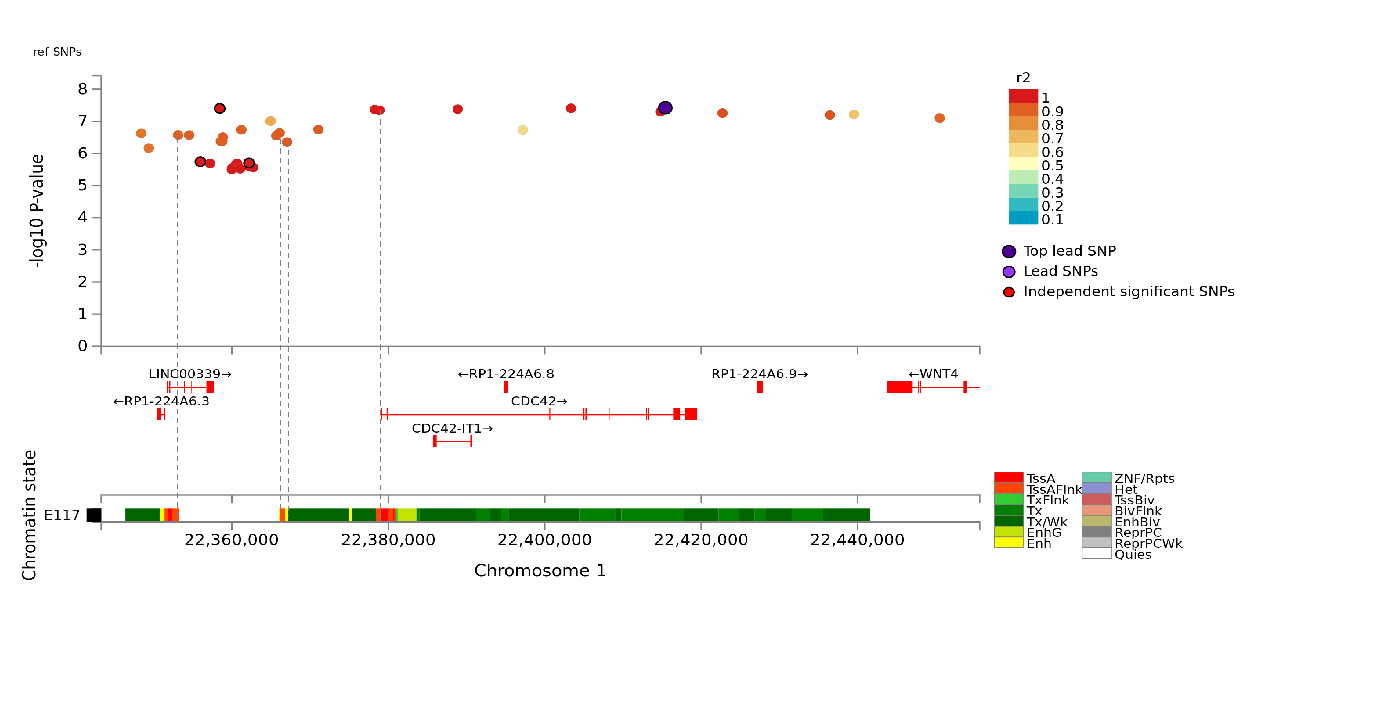 | B  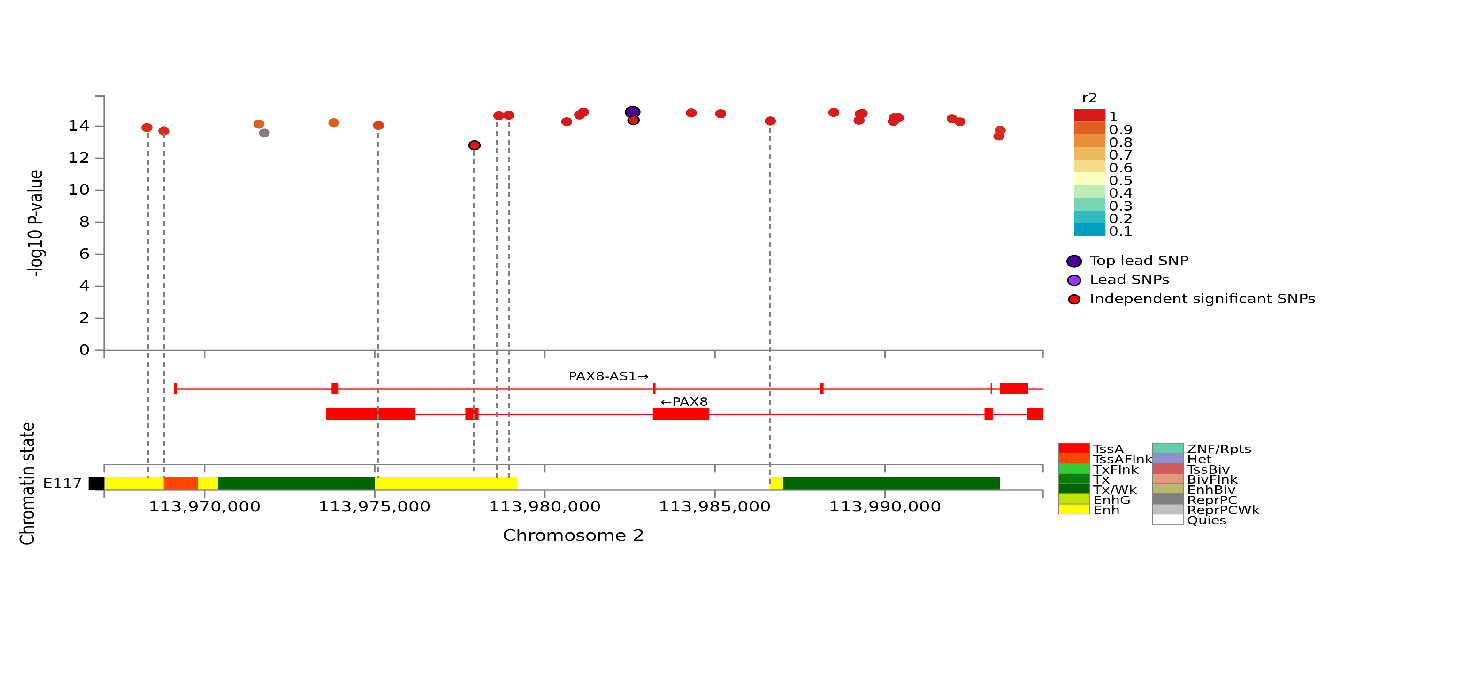 |
| --- | --- |
| C  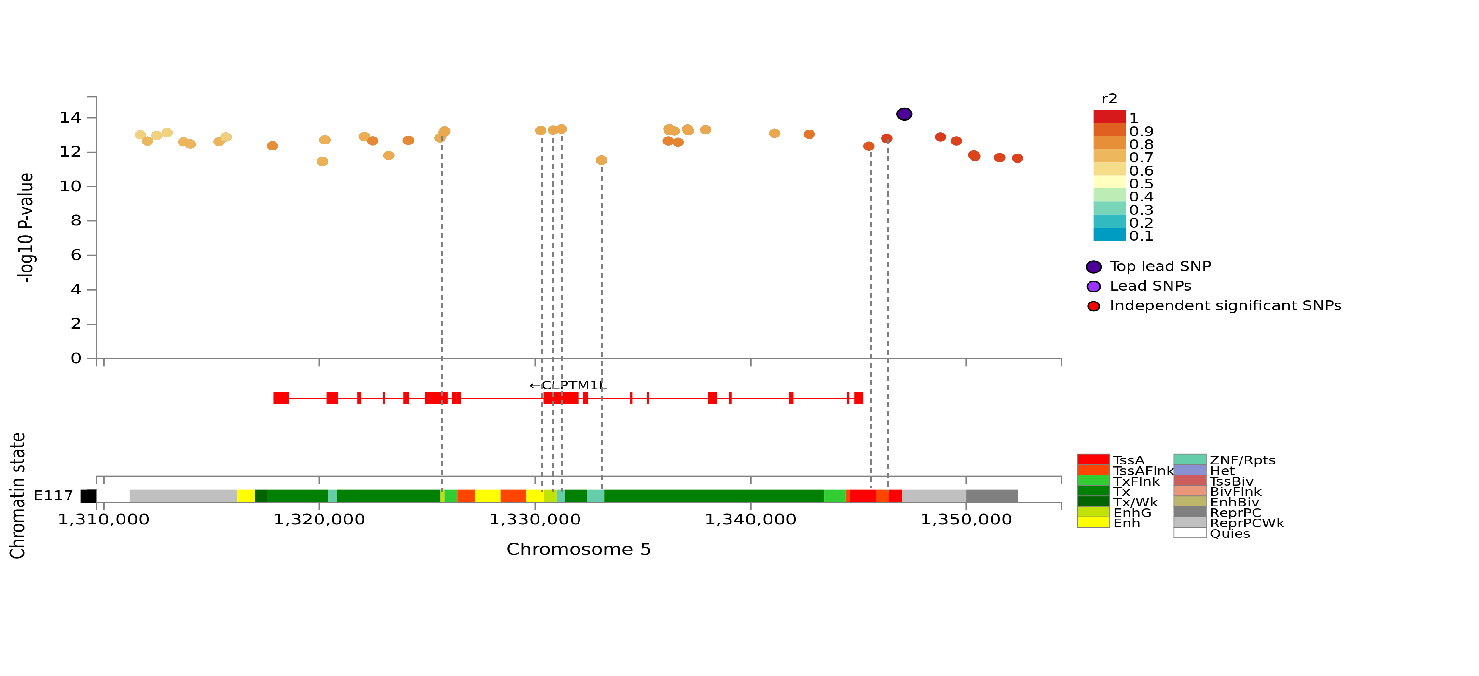 | **Supplementary Figure 4.** Cervical cancer GWAS meta-analysis credible sets for chr1 (A), chr2 (B), and chr5 (C), and their overlap with chromatin annotations in HeLa cell line (Encode Roadmap) |


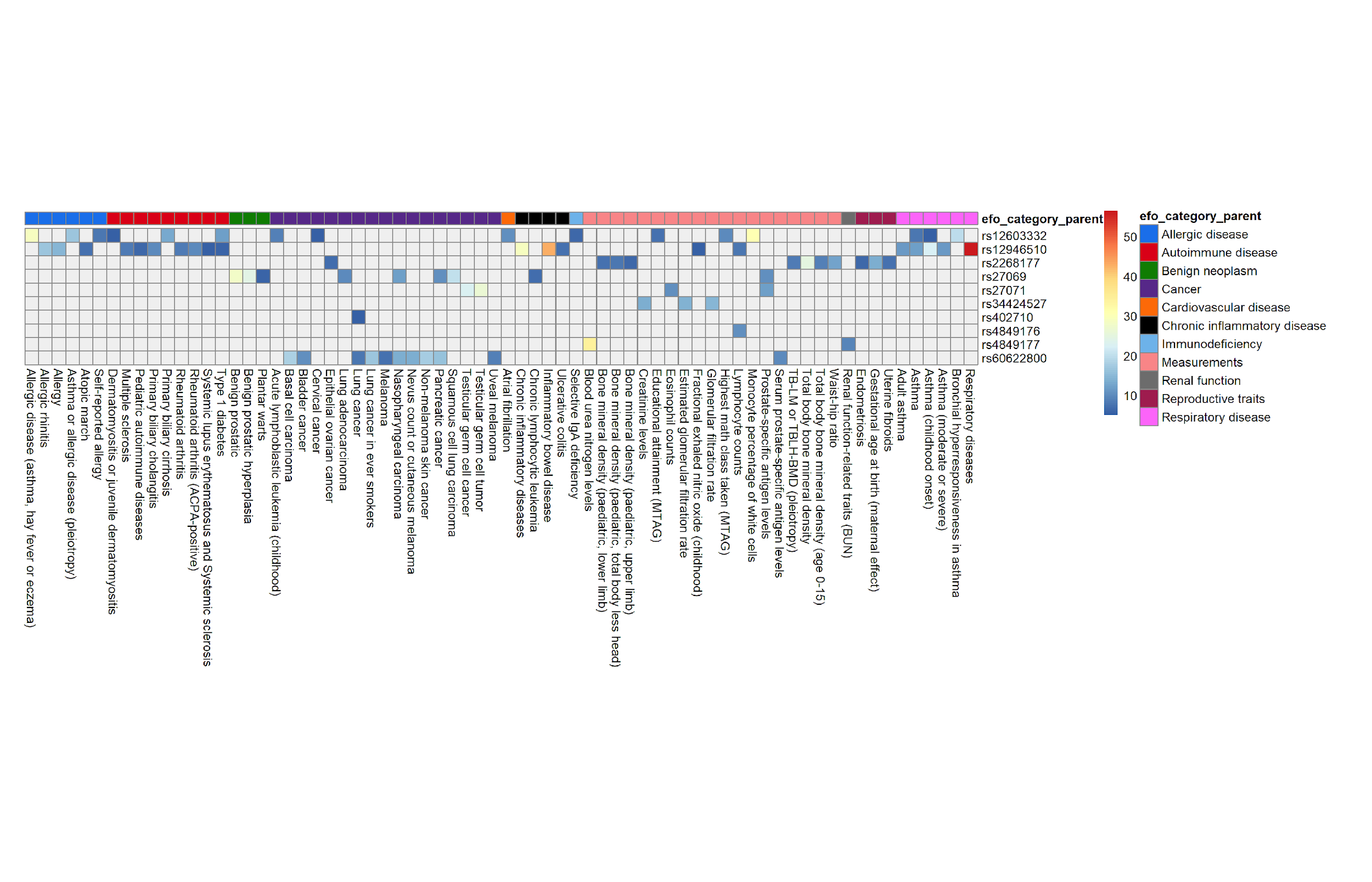
**Supplementary Figure 5**. Results of the GWAS catalog look-up for cervical cancer loci. Tiles are colored by the -log10(GWAS P-value) or grey, if there was no suggestive signal obtained for the given variant and trait (GWAS P-value > 10-5).


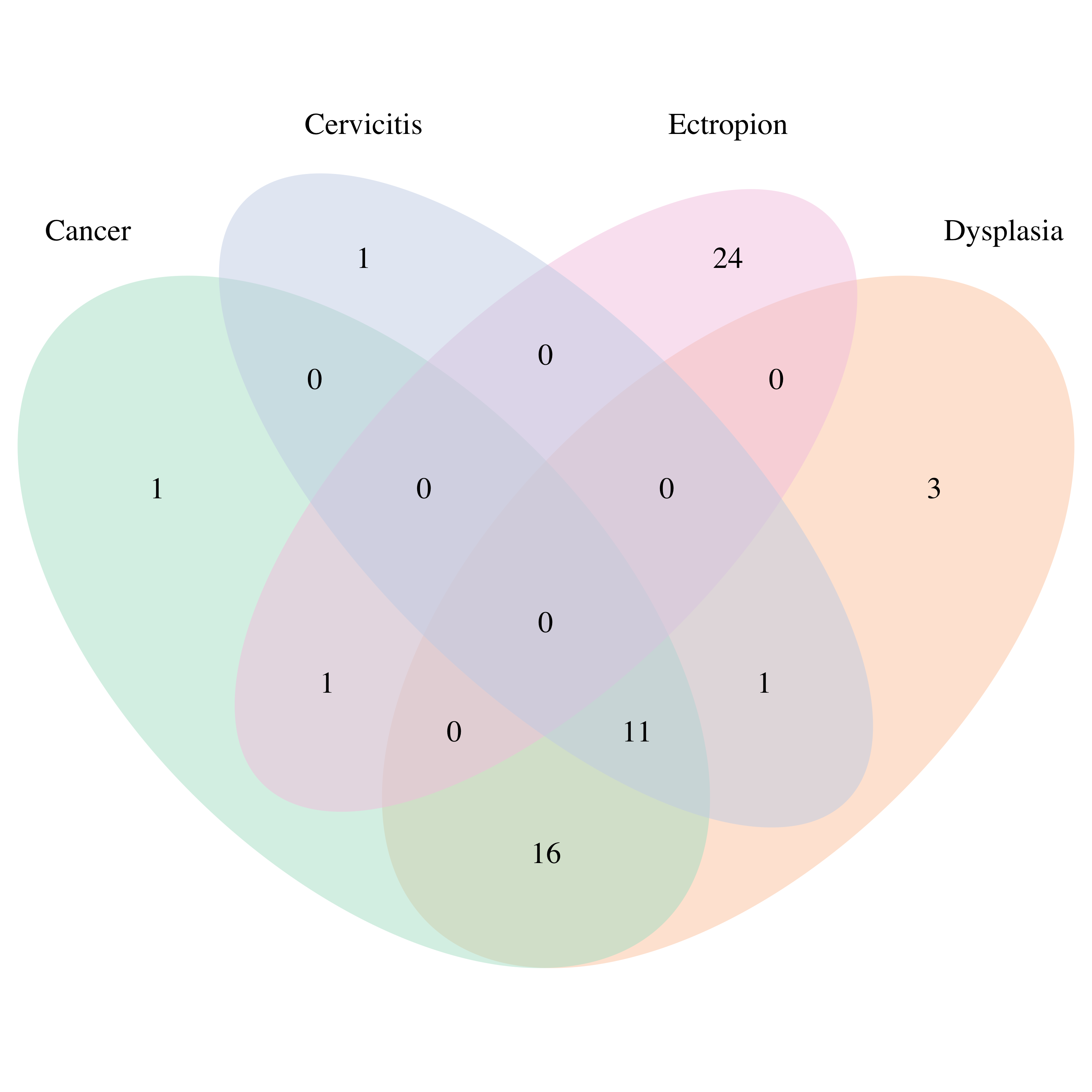


**Supplementary Figure 6.** Venn diagram illustrating the overlap between credible set SNPs in cervical ectropion, cervicitis, dysplasia and cervical cancer

| 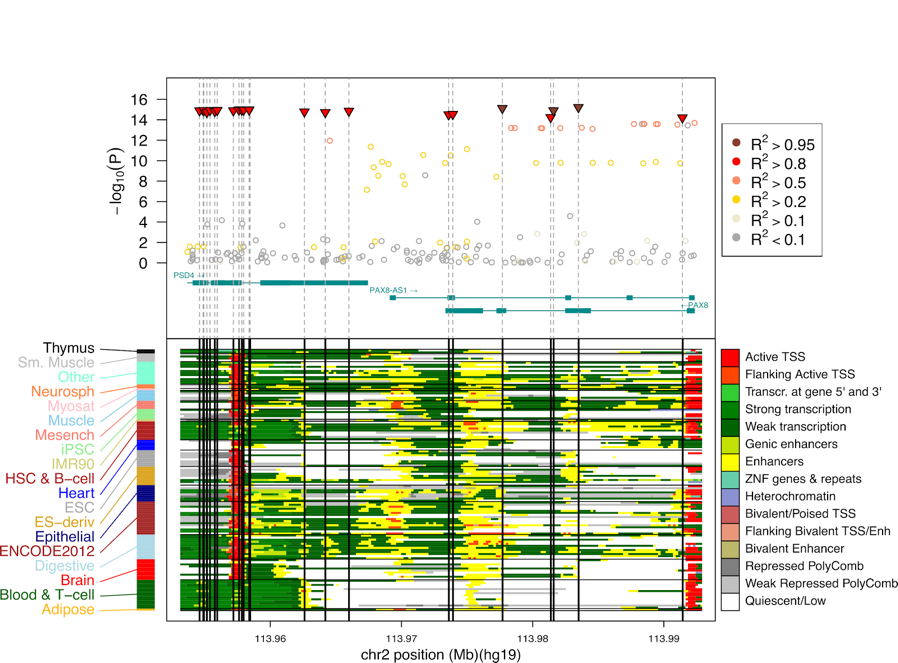  A | 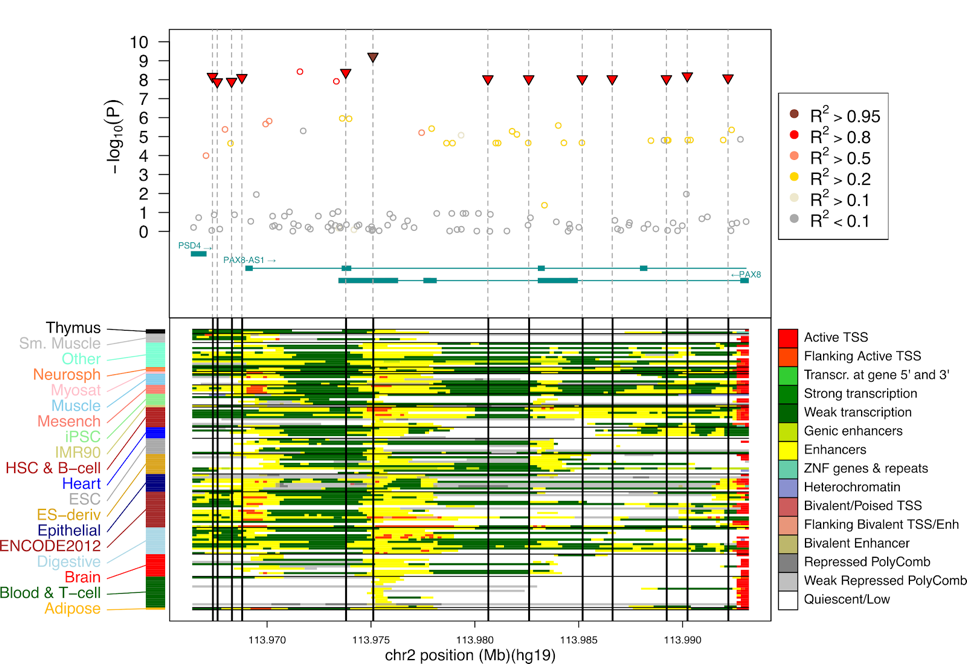  B |
| --- | --- |
| 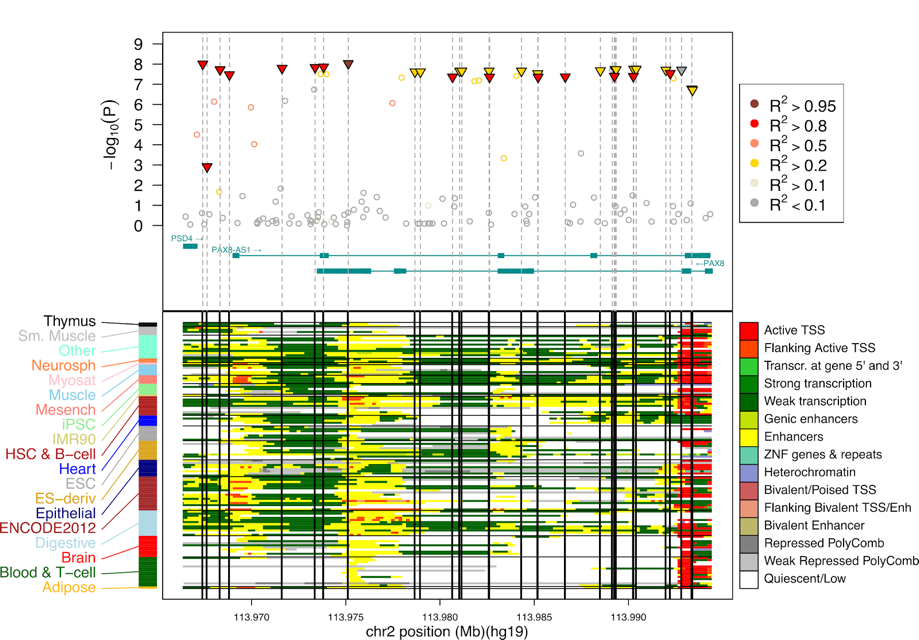C | 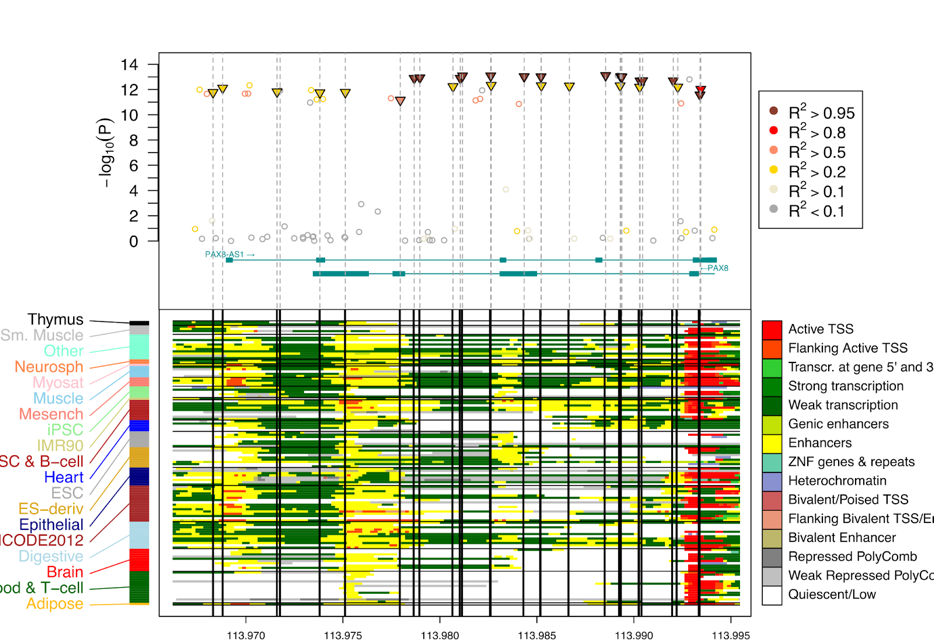D |

**Supplementary Figure 7**. Regional plots highlighting HyPrColoc credible set SNPs for chr2 locus in cervical ectropion (A), cervicitis (B), dysplasia (C) and cervical cancer (D) GWAS analysis. Bottom panel shows the corresponding Roadmap Epigenomics chromatin annotations in different tissues.


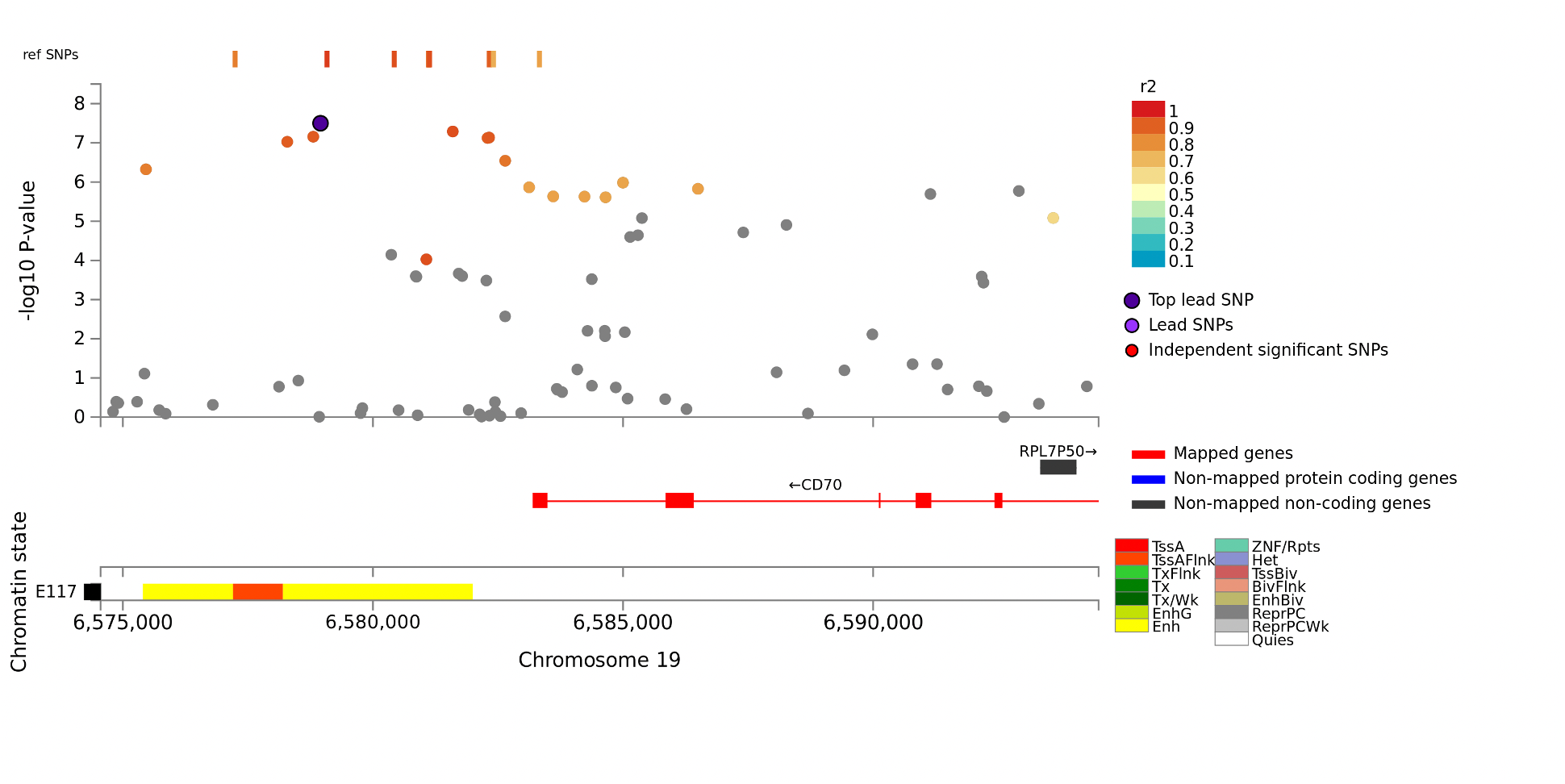


**Supplementary Figure 8.** Regional plot for locus on chromosome 19 identified in MTAG joint analysis of cervical dysplasia and cancer.


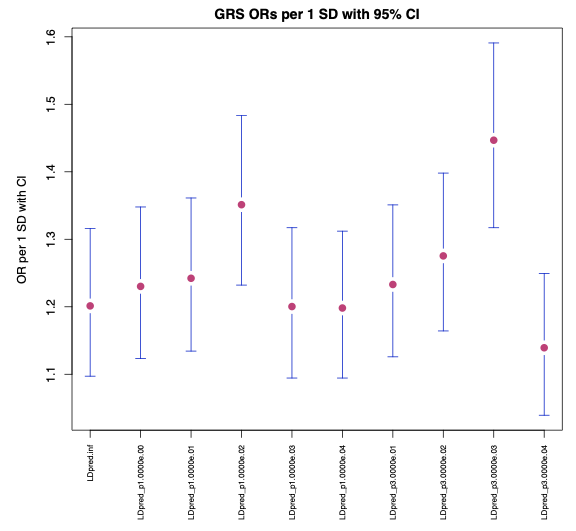


**Supplementary Figure 9.** Odds ratios (dots) and 95% CI-s (error bars) for association with cervical cancer diagnosis in discovery set for different cervical cancer genetic risk scores (x-axis).


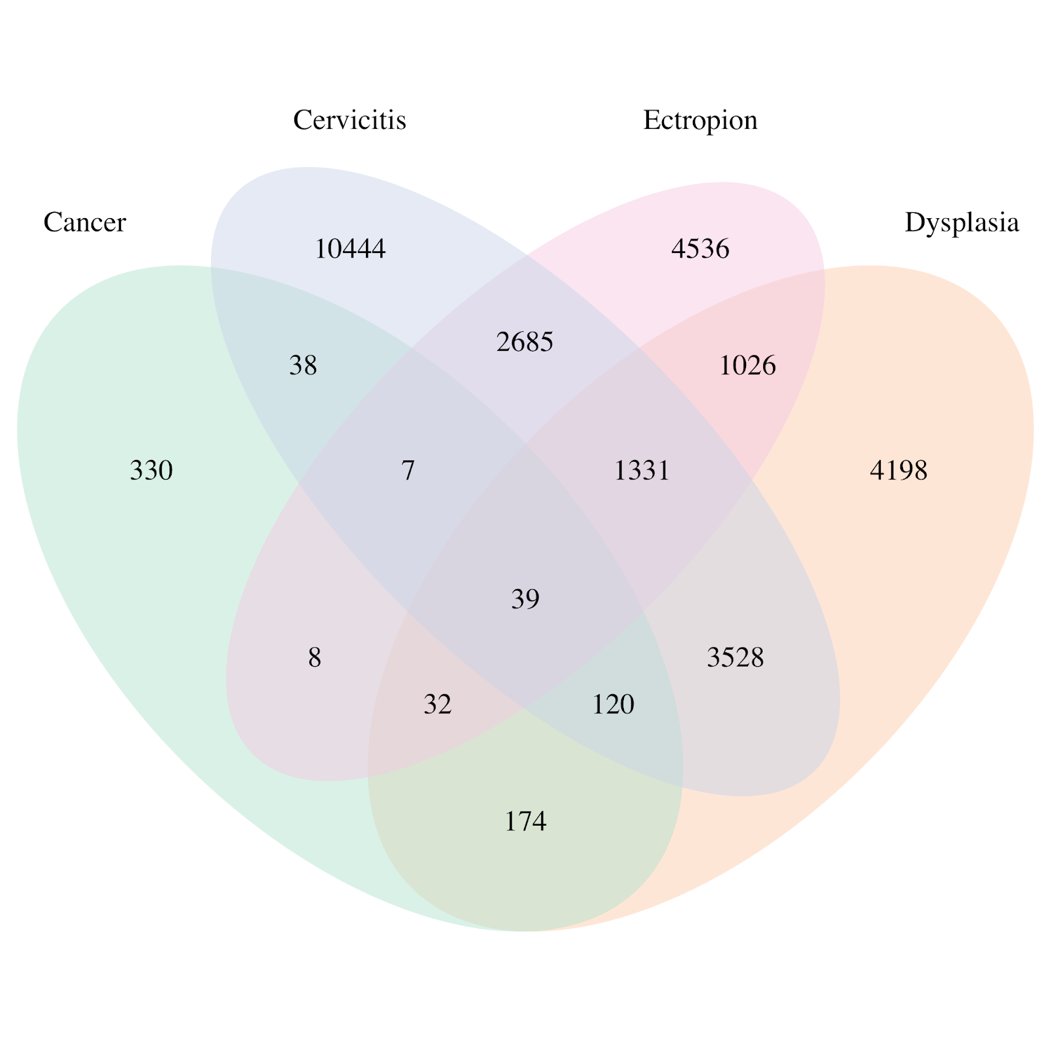


**Supplementary Figure 10**. Venn diagramm showing overlap in different diagnoses related to uterine cervix in the Estonian Biobank.


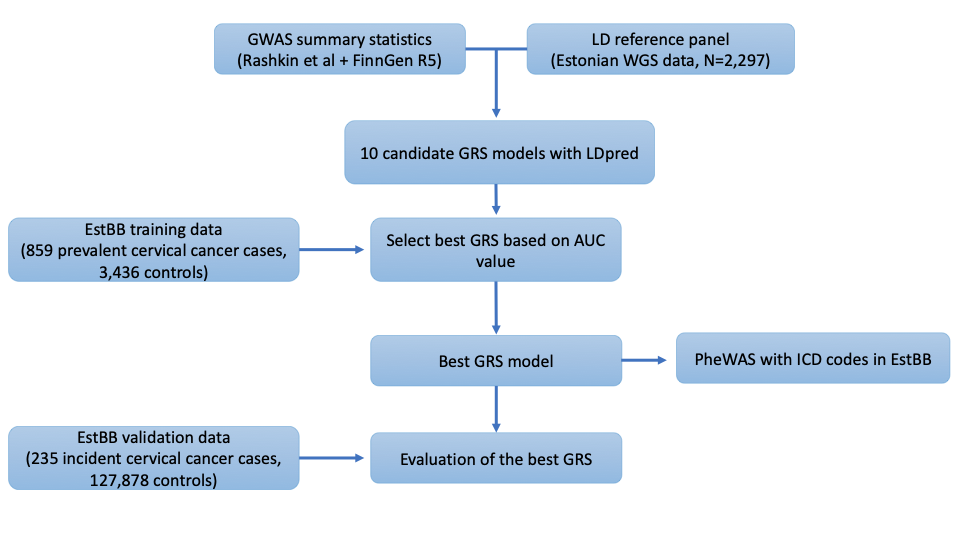


**Supplementary Figure 11**. Workflow for GRS calculation and validation in EstBB.

**Group authorships**

*Estonian Biobank Research Team*

Tõnu Esko, Andres Metspalu, Lili Milani, Mari Nelis, Georgi Hudjashov

**Contributors of FinnGen**

**Steering Committee**

Aarno Palotie Institute for Molecular Medicine Finland, HiLIFE, University of Helsinki, Finland

Mark Daly Institute for Molecular Medicine Finland, HiLIFE, University of Helsinki, Finland

**Pharmaceutical companies**

Bridget Riley-Gills Abbvie, Chicago, IL, United States

Howard Jacob Abbvie, Chicago, IL, United States

Dirk Paul Astra Zeneca, Cambridge, United Kingdom

Heiko Runz Biogen, Cambridge, MA, United States

Sally John Biogen, Cambridge, MA, United States

Robert Plenge Celgene, Summit, NJ, United States/Bristol Myers Squibb, New York, NY, United States

Mark McCarthy Genentech, San Francisco, CA, United States

Julie Hunkapiller Genentech, San Francisco, CA, United States

Meg Ehm GlaxoSmithKline, Brentford, United Kingdom

Kirsi Auro GlaxoSmithKline, Brentford, United Kingdom

Caroline Fox Merck, Kenilworth, NJ, United States

Anders Mälarstig Pfizer, New York, NY, United States

Katherine Klinger Sanofi, Paris, France

Deepak Raipal Sanofi, Paris, France

Tim Behrens Maze Therapeutics, San Francisco, CA, United States

Robert Yang Janssen Biotech, Beerse, Belgium

Richard Siegel Novartis, Basel, Switzerland

**University of Helsinki & Biobanks**

Tomi Mäkelä HiLIFE, University of Helsinki, Finland, Finland

Jaakko Kaprio Institute for Molecular Medicine Finland, HiLIFE, Helsinki, Finland, Finland

Petri Virolainen Auria Biobank / University of Turku / Hospital District of Southwest Finland, Turku, Finland

Antti Hakanen Auria Biobank / University of Turku / Hospital District of Southwest Finland, Turku, Finland

Terhi Kilpi THL Biobank / The National Institute of Health and Welfare Helsinki, Finland

Markus Perola THL Biobank / The National Institute of Health and Welfare Helsinki, Finland

Jukka Partanen Finnish Red Cross Blood Service / Finnish Hematology Registry and Clinical Biobank, Helsinki, Finland

Anne Pitkäranta Helsinki Biobank / Helsinki University and Hospital District of Helsinki and Uusimaa, Helsinki

Juhani Junttila Northern Finland Biobank Borealis / University of Oulu / Northern Ostrobothnia Hospital District, Oulu, Finland

Raisa Serpi Northern Finland Biobank Borealis / University of Oulu / Northern Ostrobothnia Hospital District, Oulu, Finland

Tarja Laitinen Finnish Clinical Biobank Tampere **/** University of Tampere / Pirkanmaa Hospital District, Tampere, Finland

Johanna Mäkelä Finnish Clinical Biobank Tampere **/** University of Tampere / Pirkanmaa Hospital District, Tampere, Finland

Veli-Matti Kosma Biobank of Eastern Finland / University of Eastern Finland / Northern Savo Hospital District, Kuopio, Finland

Urho Kujala Central Finland Biobank / University of Jyväskylä / Central Finland Health Care District, Jyväskylä, Finland

**Other Experts/ Non-Voting Members**

Outi Tuovila Business Finland, Helsinki, Finland

Raimo Pakkanen Business Finland, Helsinki, Finland

**Scientific Committee**

**Pharmaceutical companies**

Jeffrey Waring Abbvie, Chicago, IL, United States

Ali Abbasi Abbvie, Chicago, IL, United States

Mengzhen Liu Abbvie, Chicago, IL, United States

Ioanna Tachmazidou Astra Zeneca, Cambridge, United Kingdom

Chia-Yen Chen Biogen, Cambridge, MA, United States

Heiko Runz Biogen, Cambridge, MA, United States

Shameek Biswas Celgene, Summit, NJ, United States/Bristol Myers Squibb, New York, NY, United States

Julie Hunkapiller Genentech, San Francisco, CA, United States

Meg Ehm GlaxoSmithKline, Brentford, United Kingdom

Neha Raghavan Merck, Kenilworth, NJ, United States

Adriana Huertas-Vazquez Merck, Kenilworth, NJ, United States

Anders Mälarstig Pfizer, New York, NY, United States

Xinli Hu Pfizer, New York, NY, United States

Katherine Klinger Sanofi, Paris, France

Matthias Gossel Sanofi, Paris, France

Robert Graham Maze Therapeutics, San Francisco, CA, United States

Tim Behrens Maze Therapeutics, San Francisco, CA, United States

Beryl Cummings Maze Therapeutics, San Francisco, CA, United States

Wilco Fleuren Janssen Biotech, Beerse, Belgium

Dawn Waterworth Janssen Biotech, Beerse, Belgium

Nicole Renaud Novartis, Basel, Switzerland

Ma´en Obeidat Novartis, Basel, Switzerland

**University of Helsinki & Biobanks**

Samuli Ripatti Institute for Molecular Medicine Finland, HiLIFE, Helsinki, Finland

Johanna Schleutker Auria Biobank / Univ. of Turku / Hospital District of Southwest Finland, Turku, Finland

Markus Perola THL Biobank / The National Institute of Health and Welfare Helsinki, Finland

Mikko Arvas Finnish Red Cross Blood Service / Finnish Hematology Registry and Clinical Biobank, Helsinki, Finland

Olli Carpén Helsinki Biobank / Helsinki University and Hospital District of Helsinki and Uusimaa, Helsinki

Reetta Hinttala Northern Finland Biobank Borealis / University of Oulu / Northern Ostrobothnia Hospital District, Oulu, Finland

Johannes Kettunen Northern Finland Biobank Borealis / University of Oulu / Northern Ostrobothnia Hospital District, Oulu, Finland

Johanna Mäkelä Finnish Clinical Biobank Tampere **/** University of Tampere / Pirkanmaa Hospital District, Tampere, Finland

Arto Mannermaa Biobank of Eastern Finland / University of Eastern Finland / Northern Savo Hospital District, Kuopio, Finland

Jari Laukkanen Central Finland Biobank / University of Jyväskylä / Central Finland Health Care District, Jyväskylä, Finland

Urho Kujala Central Finland Biobank / University of Jyväskylä / Central Finland Health Care District, Jyväskylä, Finland

**Clinical Groups**

**Neurology Group**

Reetta Kälviäinen Northern Savo Hospital District, Kuopio, Finland

Valtteri Julkunen Northern Savo Hospital District, Kuopio, Finland

Hilkka Soininen Northern Savo Hospital District, Kuopio, Finland

Anne Remes Northern Ostrobothnia Hospital District, Oulu, Finland

Mikko Hiltunen Northern Savo Hospital District, Kuopio, Finland

Jukka Peltola Pirkanmaa Hospital District, Tampere, Finland

Pentti Tienari Hospital District of Helsinki and Uusimaa, Helsinki, Finland

Juha Rinne Hospital District of Southwest Finland, Turku, Finland

Roosa Kallionpää Hospital District of Southwest Finland, Turku, Finland

Ali Abbasi Abbvie, Chicago, IL, United States

Adam Ziemann Abbvie, Chicago, IL, United States

Jeffrey Waring Abbvie, Chicago, IL, United States

Sahar Esmaeeli Abbvie, Chicago, IL, United States

Nizar Smaoui Abbvie, Chicago, IL, United States

Anne Lehtonen Abbvie, Chicago, IL, United States

Susan Eaton Biogen, Cambridge, MA, United States

Heiko Runz Biogen, Cambridge, MA, United States

Sanni Lahdenperä Biogen, Cambridge, MA, United States

Janet van Adelsberg Celgene, Summit, NJ, United States/ Bristol Myers Squibb, New York, NY, United States

Shameek Biswas Celgene, Summit, NJ, United States/ Bristol Myers Squibb, New York, NY, United States

Julie Hunkapiller Genentech, San Francisco, CA, United States

Natalie Bowers Genentech, San Francisco, CA, United States

Edmond Teng Genentech, San Francisco, CA, United States

Sarah Pendergrass Genentech, San Francisco, CA, United States

Onuralp Soylemez Merck, Kenilworth, NJ, United States

Kari Linden Pfizer, New York, NY, United States

Fanli Xu GlaxoSmithKline, Brentford, United Kingdom

David Pulford GlaxoSmithKline, Brentford, United Kingdom

Kirsi Auro GlaxoSmithKline, Brentford, United Kingdom

Laura Addis GlaxoSmithKline, Brentford, United Kingdom

John Eicher GlaxoSmithKline, Brentford, United Kingdom

Minna Raivio Hospital District of Helsinki and Uusimaa, Helsinki, Finland

Sarah Pendergrass Genentech, San Francisco, CA, United States

Beryl Cummings Maze Therapeutics, San Francisco, CA, United States

Juulia Partanen Institute for Molecular Medicine Finland, HiLIFE, University of Helsinki, Finland

**Gastroenterology Group**

Martti Färkkilä Hospital District of Helsinki and Uusimaa, Helsinki, Finland

Jukka Koskela Hospital District of Helsinki and Uusimaa, Helsinki, Finland

Sampsa Pikkarainen Hospital District of Helsinki and Uusimaa, Helsinki, Finland

Airi Jussila Pirkanmaa Hospital District, Tampere, Finland

Katri Kaukinen Pirkanmaa Hospital District, Tampere, Finland

Timo Blomster Northern Ostrobothnia Hospital District, Oulu, Finland

Mikko Kiviniemi Northern Savo Hospital District, Kuopio, Finland

Markku Voutilainen Hospital District of Southwest Finland, Turku, Finland

Ali Abbasi Abbvie, Chicago, IL, United States

Graham Heap Abbvie, Chicago, IL, United States

Jeffrey Waring Abbvie, Chicago, IL, United States

Nizar Smaoui Abbvie, Chicago, IL, United States

Fedik Rahimov Abbvie, Chicago, IL, United States

Anne Lehtonen Abbvie, Chicago, IL, United States

Keith Usiskin Celgene, Summit, NJ, United States/ Bristol Myers Squibb, New York, NY, United States

Tim Lu Genentech, San Francisco, CA, United States

Natalie Bowers Genentech, San Francisco, CA, United States

Danny Oh Genentech, San Francisco, CA, United States

Sarah Pendergrass Genentech, San Francisco, CA, United States

Kirsi Kalpala Pfizer, New York, NY, United States

Melissa Miller Pfizer, New York, NY, United States

Xinli Hu Pfizer, New York, NY, United States

Linda McCarthy GlaxoSmithKline, Brentford, United Kingdom

Onuralp Soylemez Merck, Kenilworth, NJ, United States

Mark Daly Institute for Molecular Medicine Finland, HiLIFE, University of Helsinki, Finland

**Rheumatology Group**

Kari Eklund Hospital District of Helsinki and Uusimaa, Helsinki, Finland

Antti Palomäki Hospital District of Southwest Finland, Turku, Finland

Pia Isomäki Pirkanmaa Hospital District, Tampere, Finland

Laura Pirilä Hospital District of Southwest Finland, Turku, Finland

Oili Kaipiainen-Seppänen Northern Savo Hospital District, Kuopio, Finland

Johanna Huhtakangas Northern Ostrobothnia Hospital District, Oulu, Finland

Ali Abbasi Abbvie, Chicago, IL, United States

Jeffrey Waring Abbvie, Chicago, IL, United States

Fedik Rahimov Abbvie, Chicago, IL, United States

Apinya Lertratanakul Abbvie, Chicago, IL, United States

Nizar Smaoui Abbvie, Chicago, IL, United States

Anne Lehtonen Abbvie, Chicago, IL, United States

David Close Astra Zeneca, Cambridge, United Kingdom

Marla Hochfeld Celgene, Summit, NJ, United States/ Bristol Myers Squibb, New York, NY, United States

Natalie Bowers Genentech, San Francisco, CA, United States

Sarah Pendergrass Genentech, San Francisco, CA, United States

Onuralp Soylemez Merck, Kenilworth, NJ, United States

Kirsi Kalpala Pfizer, New York, NY, United States

Nan Bing Pfizer, New York, NY, United States

Xinli Hu Pfizer, New York, NY, United States

Jorge Esparza Gordillo GlaxoSmithKline, Brentford, United Kingdom

Kirsi Auro GlaxoSmithKline, Brentford, United Kingdom

Dawn Waterworth Janssen Biotech, Beerse, Belgium

Nina Mars Institute for Molecular Medicine Finland, HiLIFE, Helsinki, Finland

**Pulmonology Group**

Tarja Laitinen Pirkanmaa Hospital District, Tampere, Finland

Margit Pelkonen Northern Savo Hospital District, Kuopio, Finland

Paula Kauppi Hospital District of Helsinki and Uusimaa, Helsinki, Finland

Hannu Kankaanranta Pirkanmaa Hospital District, Tampere, Finland

Terttu Harju Northern Ostrobothnia Hospital District, Oulu, Finland

Riitta Lahesmaa Hospital District of Southwest Finland, Turku, Finland

Nizar Smaoui Abbvie, Chicago, IL, United States

Alex Mackay Astra Zeneca, Cambridge, United Kingdom

Glenda Lassi Astra Zeneca, Cambridge, United Kingdom

Susan Eaton Biogen, Cambridge, MA, United States

Steven Greenberg Celgene, Summit, NJ, United States/ Bristol Myers Squibb, New York, NY, United States

Hubert Chen Genentech, San Francisco, CA, United States

Sarah Pendergrass Genentech, San Francisco, CA, United States

Natalie Bowers Genentech, San Francisco, CA, United States

Joanna Betts GlaxoSmithKline, Brentford, United Kingdom

Soumitra Ghosh GlaxoSmithKline, Brentford, United Kingdom

Kirsi Auro GlaxoSmithKline, Brentford, United Kingdom

Rajashree Mishra GlaxoSmithKline, Brentford, United Kingdom

Sina Rüeger Institute for Molecular Medicine Finland, HiLIFE, University of Helsinki, Finland

**Cardiometabolic Diseases Group**

Teemu Niiranen The National Institute of Health and Welfare Helsinki, Finland

Felix Vaura The National Institute of Health and Welfare Helsinki, Finland

Veikko Salomaa The National Institute of Health and Welfare Helsinki, Finland

Markus Juonala Hospital District of Southwest Finland, Turku, Finland

Kaj Metsärinne Hospital District of Southwest Finland, Turku, Finland

Mika Kähönen Pirkanmaa Hospital District, Tampere, Finland

Juhani Junttila Northern Ostrobothnia Hospital District, Oulu, Finland

Markku Laakso Northern Savo Hospital District, Kuopio, Finland

Jussi Pihlajamäki Northern Savo Hospital District, Kuopio, Finland

Daniel Gordin Hospital District of Helsinki and Uusimaa, Helsinki, Finland

Juha Sinisalo Hospital District of Helsinki and Uusimaa, Helsinki, Finland

Marja-Riitta Taskinen Hospital District of Helsinki and Uusimaa, Helsinki, Finland

Tiinamaija Tuomi Hospital District of Helsinki and Uusimaa, Helsinki, Finland

Jari Laukkanen Central Finland Health Care District, Jyväskylä, Finland

Benjamin Challis Astra Zeneca, Cambridge, United Kingdom

Dirk Paul Astra Zeneca, Cambridge, United Kingdom

Julie Hunkapiller Genentech, San Francisco, CA, United States

Natalie Bowers Genentech, San Francisco, CA, United States

Sarah Pendergrass Genentech, San Francisco, CA, United States

Onuralp Soylemez Merck, Kenilworth, NJ, United States

Jaakko Parkkinen Pfizer, New York, NY, United States

Melissa Miller Pfizer, New York, NY, United States

Russell Miller Pfizer, New York, NY, United States

Audrey Chu GlaxoSmithKline, Brentford, United Kingdom

Kirsi Auro GlaxoSmithKline, Brentford, United Kingdom

Keith Usiskin Celgene, Summit, NJ, United States/ Bristol Myers Squibb, New York, NY, United States

Amanda Elliott Institute for Molecular Medicine Finland, HiLIFE, University of Helsinki, Finland / Broad Institute, Cambridge, MA, United States

Joel Rämö Institute for Molecular Medicine Finland, HiLIFE, University of Helsinki, Finland

Samuli Ripatti Institute for Molecular Medicine Finland, HiLIFE, University of Helsinki, Finland

Mary Pat Reeve Institute for Molecular Medicine Finland, HiLIFE, University of Helsinki, Finland

Sanni Ruotsalainen Institute for Molecular Medicine Finland, HiLIFE, University of Helsinki, Finland

**Oncology Group**

Tuomo Meretoja Hospital District of Helsinki and Uusimaa, Helsinki, Finland

Heikki Joensuu Hospital District of Helsinki and Uusimaa, Helsinki, Finland

Olli Carpén Hospital District of Helsinki and Uusimaa, Helsinki, Finland

Lauri Aaltonen Hospital District of Helsinki and Uusimaa, Helsinki, Finland

Johanna Mattson Hospital District of Helsinki and Uusimaa, Helsinki, Finland

Annika Auranen Pirkanmaa Hospital District , Tampere, Finland

Peeter Karihtala Northern Ostrobothnia Hospital District, Oulu, Finland

Saila Kauppila Northern Ostrobothnia Hospital District, Oulu, Finland

Päivi Auvinen Northern Savo Hospital District, Kuopio, Finland

Klaus Elenius Hospital District of Southwest Finland, Turku, Finland

Johanna Schleutker Hospital District of Southwest Finland, Turku, Finland

Relja Popovic Abbvie, Chicago, IL, United States

Jeffrey Waring Abbvie, Chicago, IL, United States

Bridget Riley-Gillis Abbvie, Chicago, IL, United States

Anne Lehtonen Abbvie, Chicago, IL, United States

Jennifer Schutzman Genentech, San Francisco, CA, United States

Julie Hunkapiller Genentech, San Francisco, CA, United States

Natalie Bowers Genentech, San Francisco, CA, United States

Sarah Pendergrass Genentech, San Francisco, CA, United States

Andrey Loboda Merck, Kenilworth, NJ, United States

Aparna Chhibber Merck, Kenilworth, NJ, United States

Heli Lehtonen Pfizer, New York, NY, United States

Stefan McDonough Pfizer, New York, NY, United States

Marika Crohns Sanofi, Paris, France

Sauli Vuoti Sanofi, Paris, France

Diptee Kulkarni GlaxoSmithKline, Brentford, United Kingdom

Kirsi Auro GlaxoSmithKline, Brentford, United Kingdom

Esa Pitkänen Institute for Molecular Medicine Finland, HiLIFE, University of Helsinki, Finland

Nina Mars Institute for Molecular Medicine Finland, HiLIFE, University of Helsinki, Finland

Mark Daly Institute for Molecular Medicine Finland, HiLIFE, University of Helsinki, Finland

**Opthalmology Group**

Kai Kaarniranta Northern Savo Hospital District, Kuopio, Finland

Joni A Turunen Hospital District of Helsinki and Uusimaa, Helsinki, Finland

Terhi Ollila Hospital District of Helsinki and Uusimaa, Helsinki, Finland

Sanna Seitsonen Hospital District of Helsinki and Uusimaa, Helsinki, Finland

Hannu Uusitalo Pirkanmaa Hospital District, Tampere, Finland

Vesa Aaltonen Hospital District of Southwest Finland, Turku, Finland

Hannele Uusitalo-Järvinen Pirkanmaa Hospital District, Tampere, Finland

Marja Luodonpää Northern Ostrobothnia Hospital District, Oulu, Finland

Nina Hautala Northern Ostrobothnia Hospital District, Oulu, Finland

Mengzhen Liu Abbvie, Chicago, IL, United States

Heiko Runz Biogen, Cambridge, MA, United States

Stephanie Loomis Biogen, Cambridge, MA, United States

Erich Strauss Genentech, San Francisco, CA, United States

Natalie Bowers Genentech, San Francisco, CA, United States

Hao Chen Genentech, San Francisco, CA, United States

Sarah Pendergrass Genentech, San Francisco, CA, United States

Anna Podgornaia Merck, Kenilworth, NJ, United States

Juha Karjalainen Institute for Molecular Medicine Finland, HiLIFE, University of Helsinki, Finland / Broad Institute, Cambridge, MA, United States

Esa Pitkänen Institute for Molecular Medicine Finland, HiLIFE, University of Helsinki, Finland

**Dermatology Group**

Kaisa Tasanen Northern Ostrobothnia Hospital District, Oulu, Finland

Laura Huilaja Northern Ostrobothnia Hospital District, Oulu, Finland

Katariina Hannula-Jouppi Hospital District of Helsinki and Uusimaa, Helsinki, Finland

Teea Salmi Pirkanmaa Hospital District, Tampere, Finland

Sirkku Peltonen Hospital District of Southwest Finland, Turku, Finland

Leena Koulu Hospital District of Southwest Finland, Turku, Finland

Kirsi Kalpala Pfizer, New York, NY, United States

Ying Wu Pfizer, New York, NY, United States

David Choy Genentech, San Francisco, CA, United States

Sarah Pendergrass Genentech, San Francisco, CA, United States

Nizar Smaoui Abbvie, Chicago, IL, United States

Fedik Rahimov Abbvie, Chicago, IL, United States

Anne Lehtonen Abbvie, Chicago, IL, United States

Dawn Waterworth Janssen Biotech, Beerse, Belgium

**Odontology Group**

Pirkko Pussinen Hospital District of Helsinki and Uusimaa, Helsinki, Finland

Aino Salminen Hospital District of Helsinki and Uusimaa, Helsinki, Finland

Tuula Salo Hospital District of Helsinki and Uusimaa, Helsinki, Finland

David Rice Hospital District of Helsinki and Uusimaa, Helsinki, Finland

Pekka Nieminen Hospital District of Helsinki and Uusimaa, Helsinki, Finland

Ulla Palotie Hospital District of Helsinki and Uusimaa, Helsinki, Finland

Juha Sinisalo Hospital District of Helsinki and Uusimaa, Helsinki, Finland

Maria Siponen Northern Savo Hospital District, Kuopio, Finland

Liisa Suominen Northern Savo Hospital District, Kuopio, Finland

Päivi Mäntylä Northern Savo Hospital District, Kuopio, Finland

Ulvi Gursoy Hospital District of Southwest Finland, Turku, Finland

Vuokko Anttonen Northern Ostrobothnia Hospital District, Oulu, Finland

Kirsi Sipilä Northern Ostrobothnia Hospital District, Oulu, Finland

Sarah Pendergrass Genentech, San Francisco, CA, United States

**Women’s Health and Reproduction Group**

Hannele Laivuori Institute for Molecular Medicine Finland, HiLIFE, University of Helsinki, Finland

Venla Kurra Pirkanmaa Hospital District, Tampere, Finland

Oskari Heikinheimo Hospital District of Helsinki and Uusimaa, Helsinki, Finland

Ilkka Kalliala Hospital District of Helsinki and Uusimaa, Helsinki, Finland

Laura Kotaniemi-Talonen Pirkanmaa Hospital District, Tampere, Finland

Kari Nieminen Pirkanmaa Hospital District, Tampere, Finland

Päivi Polo Hospital District of Southwest Finland, Turku, Finland

Kaarin Mäkikallio Hospital District of Southwest Finland, Turku, Finland

Eeva Ekholm Hospital District of Southwest Finland, Turku, Finland

Marja Vääräsmäki Northern Ostrobothnia Hospital District, Oulu, Finland

Outi Uimari Northern Ostrobothnia Hospital District, Oulu, Finland

Laure Morin-Papunen Northern Ostrobothnia Hospital District, Oulu, Finland

Marjo Tuppurainen Northern Savo Hospital District, Kuopio, Finland

Katja Kivinen Institute for Molecular Medicine Finland, HiLIFE, University of Helsinki, Finland

Elisabeth Widen Institute for Molecular Medicine Finland, HiLIFE, University of Helsinki, Finland

Taru Tukiainen Institute for Molecular Medicine Finland, HiLIFE, University of Helsinki, Finland

Mary Pat Reeve Institute for Molecular Medicine Finland, HiLIFE, University of Helsinki, Finland

Mark Daly Institute for Molecular Medicine Finland, HiLIFE, University of Helsinki, Finland

Liu Aoxing Institute for Molecular Medicine Finland, HiLIFE, University of Helsinki, Finland

Eija Laakkonen University of Jyväskylä, Jyväskylä, Finland

Niko Välimäki University of Helsinki, Helsinki, Finland

Lauri Aaltonen Hospital District of Helsinki and Uusimaa, Helsinki, Finland

Johannes Kettunen Northern Ostrobothnia Hospital District, Oulu, Finland

Mikko Arvas Finnish Red Cross Blood Service, Helsinki, Finland

Jeffrey Waring Abbvie, Chicago, IL, United States

Bridget Riley-Gillis Abbvie, Chicago, IL, United States

Mengzhen Liu Abbvie, Chicago, IL, United States

Janet Kumar GlaxoSmithKline, Brentford, United Kingdom

Kirsi Auro GlaxoSmithKline, Brentford, United Kingdom

Andrea Ganna Institute for Molecular Medicine Finland, HiLIFE, University of Helsinki, Finland

Sarah Pendergrass Genentech, San Francisco, CA, United States

**FinnGen Analysis working group**

Justin Wade Davis Abbvie, Chicago, IL, United States

Bridget Riley-Gillis Abbvie, Chicago, IL, United States

Danjuma Quarless Abbvie, Chicago, IL, United States

Fedik Rahimov Abbvie, Chicago, IL, United States

Sahar Esmaeeli Abbvie, Chicago, IL, United States

Slavé Petrovski Astra Zeneca, Cambridge, United Kingdom

Eleonor Wigmore Astra Zeneca, Cambridge, United Kingdom

Adele Mitchell Biogen, Cambridge, MA, United States

Benjamin Sun Biogen, Cambridge, MA, United States

Ellen Tsai Biogen, Cambridge, MA, United States

Denis Baird Biogen, Cambridge, MA, United States

Paola Bronson Biogen, Cambridge, MA, United States

Ruoyu Tian Biogen, Cambridge, MA, United States

Stephanie Loomis Biogen, Cambridge, MA, United States

Yunfeng Huang Biogen, Cambridge, MA, United States

Joseph Maranville Celgene, Summit, NJ, United States/ Bristol Myers Squibb, New York, NY, United States

Shameek Biswas Celgene, Summit, NJ, United States/ Bristol Myers Squibb, New York, NY, United States

Elmutaz Mohammed Celgene, Summit, NJ, United States/ Bristol Myers Squibb, New York, NY, United States

Samir Wadhawan Celgene, Summit, NJ, United States/ Bristol Myers Squibb, New York, NY, United States

Erika Kvikstad Celgene, Summit, NJ, United States/ Bristol Myers Squibb, New York, NY, United States

Minal Caliskan Celgene, Summit, NJ, United States/ Bristol Myers Squibb, New York, NY, United States

Diana Chang Genentech, San Francisco, CA, United States

Julie Hunkapiller Genentech, San Francisco, CA, United States

Tushar Bhangale Genentech, San Francisco, CA, United States

Natalie Bowers Genentech, San Francisco, CA, United States

Sarah Pendergrass Genentech, San Francisco, CA, United States

Kirill Shkura Merck, Kenilworth, NJ, United States

Victor Neduva Merck, Kenilworth, NJ, United States

Xing Chen Pfizer, New York, NY, United States

Åsa Hedman Pfizer, New York, NY, United States

Karen S King GlaxoSmithKline, Brentford, United Kingdom

Padhraig Gormley GlaxoSmithKline, Brentford, United Kingdom

Jimmy Liu GlaxoSmithKline, Brentford, United Kingdom

Clarence Wang Sanofi, Paris, France

Ethan Xu Sanofi, Paris, France

Franck Auge Sanofi, Paris, France

Clement Chatelain Sanofi, Paris, France

Deepak Rajpal Sanofi, Paris, France

Dongyu Liu Sanofi, Paris, France

Katherine Call Sanofi, Paris, France

Tai-He Xia Sanofi, Paris, France

Beryl Cummings Maze Therapeutics, San Francisco, CA, United States

Matt Brauer Maze Therapeutics, San Francisco, CA, United States

Huilei Xu Novartis, Basel, Switzerland

Amy Cole Novartis, Basel, Switzerland

Jonathan Chung Novartis, Basel, Switzerland

Jaison Jacob Novartis, Basel, Switzerland

Katrina de Lange Novartis, Basel, Switzerland

Jonas Zierer Novartis, Basel, Switzerland

Mitja Kurki Institute for Molecular Medicine Finland, HiLIFE, University of Helsinki, Finland / Broad Institute, Cambridge, MA, United States

Samuli Ripatti Institute for Molecular Medicine Finland, HiLIFE, University of Helsinki, Finland

Mark Daly Institute for Molecular Medicine Finland, HiLIFE, University of Helsinki, Finland

Juha Karjalainen Institute for Molecular Medicine Finland, HiLIFE, University of Helsinki, Finland / Broad Institute, Cambridge, MA, United States

Aki Havulinna Institute for Molecular Medicine Finland, HiLIFE, University of Helsinki, Finland

Juha Mehtonen Institute for Molecular Medicine Finland, HiLIFE, University of Helsinki, Finland

Priit Palta Institute for Molecular Medicine Finland, HiLIFE, University of Helsinki, Finland

Shabbeer Hassan Institute for Molecular Medicine Finland, HiLIFE, University of Helsinki, Finland

Pietro Della Briotta Parolo Institute for Molecular Medicine Finland, HiLIFE, University of Helsinki, Finland

Wei Zhou Broad Institute, Cambridge, MA, United States

Mutaamba Maasha Broad Institute, Cambridge, MA, United States

Shabbeer Hassan Institute for Molecular Medicine Finland, HiLIFE, University of Helsinki, Finland

Susanna Lemmelä Institute for Molecular Medicine Finland, HiLIFE, University of Helsinki, Finland

Manuel Rivas University of Stanford, Stanford, CA, United States

Aarno Palotie Institute for Molecular Medicine Finland, HiLIFE, University of Helsinki, Finland

Arto Lehisto Institute for Molecular Medicine Finland, HiLIFE, University of Helsinki, Finland

Andrea Ganna Institute for Molecular Medicine Finland, HiLIFE, University of Helsinki, Finland

Vincent Llorens Institute for Molecular Medicine Finland, HiLIFE, University of Helsinki, Finland

Hannele Laivuori Institute for Molecular Medicine Finland, HiLIFE, University of Helsinki, Finland

Mari E Niemi Institute for Molecular Medicine Finland, HiLIFE, University of Helsinki, Finland

Taru Tukiainen Institute for Molecular Medicine Finland, HiLIFE, University of Helsinki, Finland

Mary Pat Reeve Institute for Molecular Medicine Finland, HiLIFE, University of Helsinki, Finland

Henrike Heyne Institute for Molecular Medicine Finland, HiLIFE, University of Helsinki, Finland

Nina Mars Institute for Molecular Medicine Finland, HiLIFE, University of Helsinki, Finland

Kimmo Palin University of Helsinki, Helsinki, Finland

Javier Garcia-Tabuenca University of Tampere, Tampere, Finland

Harri Siirtola University of Tampere, Tampere, Finland

Tuomo Kiiskinen Institute for Molecular Medicine Finland, HiLIFE, University of Helsinki, Finland

Jiwoo Lee Institute for Molecular Medicine Finland, HiLIFE, University of Helsinki, Finland / Broad Institute, Cambridge, MA, United States

Kristin Tsuo Institute for Molecular Medicine Finland, HiLIFE, University of Helsinki, Finland / Broad Institute, Cambridge, MA, United States

Amanda Elliott Institute for Molecular Medicine Finland, HiLIFE, University of Helsinki, Finland / Broad Institute, Cambridge, MA, United States

Kati Kristiansson THL Biobank / The National Institute of Health and Welfare Helsinki, Finland

Mikko Arvas Finnish Red Cross Blood Service / Finnish Hematology Registry and Clinical Biobank, Helsinki, Finland

Kati Hyvärinen Finnish Red Cross Blood Service, Helsinki, Finland

Jarmo Ritari Finnish Red Cross Blood Service, Helsinki, Finland

Miika Koskinen Helsinki Biobank / Helsinki University and Hospital District of Helsinki and Uusimaa, Helsinki

Olli Carpén Helsinki Biobank / Helsinki University and Hospital District of Helsinki and Uusimaa, Helsinki

Johannes Kettunen Northern Finland Biobank Borealis / University of Oulu / Northern Ostrobothnia Hospital District, Oulu, Finland

Katri Pylkäs University of Oulu, Oulu, Finland

Marita Kalaoja University of Oulu, Oulu, Finland

Minna Karjalainen University of Oulu, Oulu, Finland

Tuomo Mantere Northern Finland Biobank Borealis / University of Oulu / Northern Ostrobothnia Hospital District, Oulu, Finland

Eeva Kangasniemi Finnish Clinical Biobank Tampere **/** University of Tampere / Pirkanmaa Hospital District, Tampere, Finland

Sami Heikkinen University of Eastern Finland, Kuopio, Finland

Arto Mannermaa Biobank of Eastern Finland / University of Eastern Finland / Northern Savo Hospital District, Kuopio, Finland

Eija Laakkonen University of Jyväskylä, Jyväskylä, Finland

Samuel Heron University of Turku, Turku, Finland

Dhanaprakash Jambulingam University of Turku, Turku, Finland

Venkat Subramaniam Rathinakannan University of Turku, Turku, Finland

Nina Pitkänen Auria Biobank / University of Turku / Hospital District of Southwest Finland, Turku, Finland

**Biobank directors**

Lila Kallio Auria Biobank / University of Turku / Hospital District of Southwest Finland, Turku, Finland

Sirpa Soini THL Biobank / The National Institute of Health and Welfare Helsinki, Finland

Jukka Partanen Finnish Red Cross Blood Service / Finnish Hematology Registry and Clinical Biobank, Helsinki, Finland

Eero Punkka Helsinki Biobank / Helsinki University and Hospital District of Helsinki and Uusimaa, Helsinki

Raisa Serpi Northern Finland Biobank Borealis / University of Oulu / Northern Ostrobothnia Hospital District, Oulu, Finland

Johanna Mäkelä Finnish Clinical Biobank Tampere **/** University of Tampere / Pirkanmaa Hospital District, Tampere, Finland

Veli-Matti Kosma Biobank of Eastern Finland / University of Eastern Finland / Northern Savo Hospital District, Kuopio, Finland

Teijo Kuopio Central Finland Biobank / University of Jyväskylä / Central Finland Health Care District, Jyväskylä, Finland

**FinnGen Teams**

**Administration**

Anu Jalanko Institute for Molecular Medicine Finland, HiLIFE, University of Helsinki, Finland

Huei-Yi Shen Institute for Molecular Medicine Finland, HiLIFE, University of Helsinki, Finland

Risto Kajanne Institute for Molecular Medicine Finland, HiLIFE, University of Helsinki, Finland

Mervi Aavikko Institute for Molecular Medicine Finland, HiLIFE, University of Helsinki, Finland

**Analysis**

Mitja Kurki Institute for Molecular Medicine Finland, HiLIFE, University of Helsinki, Finland / Broad Institute, Cambridge, MA, United States

Juha Karjalainen Institute for Molecular Medicine Finland, HiLIFE, University of Helsinki, Finland / Broad Institute, Cambridge, MA, United States

Pietro Della Briotta Parolo Institute for Molecular Medicine Finland, HiLIFE, University of Helsinki, Finland

Arto Lehisto Institute for Molecular Medicine Finland, HiLIFE, University of Helsinki, Finland

Juha Mehtonen Institute for Molecular Medicine Finland, HiLIFE, University of Helsinki, Finland

Wei Zhou Broad Institute, Cambridge, MA, United States

Masahiro Kanai Broad Institute, Cambridge, MA, United States

Mutaamba Maasha Broad Institute, Cambridge, MA, United States

**Clinical Endpoint Development**

Hannele Laivuori Institute for Molecular Medicine Finland, HiLIFE, University of Helsinki, Finland

Aki Havulinna Institute for Molecular Medicine Finland, HiLIFE, University of Helsinki, Finland

Susanna Lemmelä Institute for Molecular Medicine Finland, HiLIFE, University of Helsinki, Finland

Tuomo Kiiskinen Institute for Molecular Medicine Finland, HiLIFE, University of Helsinki, Finland

L. Elisa Lahtela Institute for Molecular Medicine Finland, HiLIFE, University of Helsinki, Finland

**Communication**

Mari Kaunisto Institute for Molecular Medicine Finland, HiLIFE, University of Helsinki, Finland

**E-Science**

Elina Kilpeläinen Institute for Molecular Medicine Finland, HiLIFE, University of Helsinki, Finland

Timo P. Sipilä Institute for Molecular Medicine Finland, HiLIFE, University of Helsinki, Finland

Georg Brein Institute for Molecular Medicine Finland, HiLIFE, University of Helsinki, Finland

Oluwaseun Alexander Dada Institute for Molecular Medicine Finland, HiLIFE, University of Helsinki, Finland

Awaisa Ghazal Institute for Molecular Medicine Finland, HiLIFE, University of Helsinki, Finland

Anastasia Shcherban Institute for Molecular Medicine Finland, HiLIFE, University of Helsinki, Finland

**Genotyping**

Kati Donner Institute for Molecular Medicine Finland, HiLIFE, University of Helsinki, Finland

Timo P. Sipilä Institute for Molecular Medicine Finland, HiLIFE, University of Helsinki, Finland

**Sample Collection Coordination**

Anu Loukola Helsinki Biobank / Helsinki University and Hospital District of Helsinki and Uusimaa, Helsinki

**Sample Logistics**

Päivi Laiho THL Biobank / The National Institute of Health and Welfare Helsinki, Finland

Tuuli Sistonen THL Biobank / The National Institute of Health and Welfare Helsinki, Finland

Essi Kaiharju THL Biobank / The National Institute of Health and Welfare Helsinki, Finland

Markku Laukkanen THL Biobank / The National Institute of Health and Welfare Helsinki, Finland

Elina Järvensivu THL Biobank / The National Institute of Health and Welfare Helsinki, Finland

Sini Lähteenmäki THL Biobank / The National Institute of Health and Welfare Helsinki, Finland

Lotta Männikkö THL Biobank / The National Institute of Health and Welfare Helsinki, Finland

Regis Wong THL Biobank / The National Institute of Health and Welfare Helsinki, Finland

**Registry Data Operations**

Hannele Mattsson THL Biobank / The National Institute of Health and Welfare Helsinki, Finland

Kati Kristiansson THL Biobank / The National Institute of Health and Welfare Helsinki, Finland

Susanna Lemmelä Institute for Molecular Medicine Finland, HiLIFE, University of Helsinki, Finland

Sami Koskelainen THL Biobank / The National Institute of Health and Welfare Helsinki, Finland

Tero Hiekkalinna THL Biobank / The National Institute of Health and Welfare Helsinki, Finland

Teemu Paajanen THL Biobank / The National Institute of Health and Welfare Helsinki, Finland

**Sequencing Informatics**

Priit Palta Institute for Molecular Medicine Finland, HiLIFE, University of Helsinki, Finland

Kalle Pärn Institute for Molecular Medicine Finland, HiLIFE, University of Helsinki, Finland

Shuang Luo Institute for Molecular Medicine Finland, HiLIFE, University of Helsinki, Finland

Vishal Sinha Institute for Molecular Medicine Finland, HiLIFE, University of Helsinki, Finland

**Trajectory**

Tarja Laitinen Pirkanmaa Hospital District, Tampere, Finland

Mary Pat Reeve Institute for Molecular Medicine Finland, HiLIFE, University of Helsinki, Finland

Harri Siirtola University of Tampere, Tampere, Finland

Javier Gracia-Tabuenca University of Tampere, Tampere, Finland

Mika Helminen University of Tampere, Tampere, Finland

Tiina Luukkaala University of Tampere, Tampere, Finland

Iida Vähätalo University of Tampere, Tampere, Finland

**Data protection officer**

Tero Jyrhämä Institute for Molecular Medicine Finland, HiLIFE, University of Helsinki, Finland

**FinBB - Finnish biobank cooperative**

Marco Hautalahti

Laura Mustaniemi

Mirkka Koivusalo

Sarah Smith

Tom Southerington
